# Fluid drawing printing 3D conductive structures for flexible circuit manufacturing

**DOI:** 10.1038/s41378-025-00936-0

**Published:** 2025-05-12

**Authors:** Yikang Li, Dazhi Wang, Yiwen Feng, Xiangji Chen, Xu Chen, Chang Liu, Yanteng Li, Liujia Suo, Ran Zhang, Xiaopeng Zhang, Ben Liu, Fengshu Wang, Shiwen Liang, Lingjie Kong, Qiang Fu, Tongqun Ren, Tiesheng Wang

**Affiliations:** 1 Key Laboratory for Micro/Nano Technology and System of Liaoning Province, Dalian University of Technology, 116024 Dalian, China; 2 State Key Laboratory of High-performance Precision Manufacturing, Dalian University of Technology, 116024 Dalian, China; 3 Liaoning Huanghai Laboratory, 116024 Dalian, China; 4 Ningbo Institute of Dalian University of Technology, 315000 Ningbo, China; 5 State Key Laboratory of Structural Analysis for Industrial Equipment, Dalian University of Technology, 116024 Dalian, China; 6 Ningbo Sunny Opotech Co., Ltd, 315400 Ningbo, China

**Keywords:** Electrical and electronic engineering, Nanoparticles

## Abstract

Three-dimensional (3D) conductive structures significantly reduce flexible circuit complexity and enhance circuit integration. Direct extrusion printing technology offers the advantages of various material applicability and high flexibility for fabricating filamentary interconnects. The printing resolution is, however, highly dependent on the needle size. A micro-printing method was proposed based on fluid drawing to fabricate freestanding 3D conductive structures. The delicate structure is drawn out under the tension when printing. The printing material is a high-viscosity ink composed of silver nanoparticles (AgNPs) and polyvinylpyrrolidone (PVP). The viscosity is controlled by evaporating the ink’s solvent for drawing prints. This unique printing method utilizes a single needle, controlled by precise air pressure and speed, to construct 3D filamentary structures with varied wire widths. The 3D conductive structures exhibit superior structural retention and enhanced conductivity by thermal treatment. The drawing printing method has been successfully implemented on flexible circuits, including light-emitting diode (LED) arrays, thermal imaging displays, and multivibrator circuits. This work establishes a novel paradigm for flexible electronics manufacturing through fluid-drawing printing, achieving unprecedented customization and compatibility in fabricating 3D interconnects.

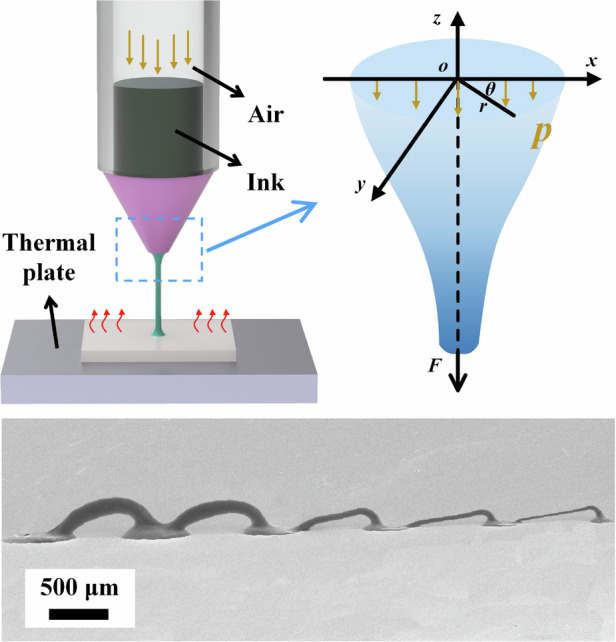

## Introduction

Printed electronics technology has received widespread attention with the advantages of low processing temperature, avoiding etching, and personalized preparation^1–3^. In addition, it has a wide range of applications for fabricating electronic devices on rigid and flexible substrates^4,5^. Numerous technologies have been developed for printed electronics. The field of printed electronics technology has developed various printing techniques, including inkjet^6^, gravure^7^, and screen printing^8^. As device density within flexible circuits gradually increases, the design complexity of planar conductive paths also grows exponentially with it^9,10^. However, conventional printing methods are limited to producing only 2D and low-aspect-ratio structures, making them unsuitable for the manufacturing needs of higher-density circuits^11–13^. 3D conductive pathways provide solutions for intricate circuit designs but face processing complexity and cost challenges in their implementation^14–17^.

Direct writing printing technology is ideal for fabricating simple, filamentary, omnidirectional 3D conductive structures because of its high compatibility with many materials, fast prototypes, mild processing conditions, and flexible manufacturing^18–21^. Direct write printing technology primarily utilizes extrusion printing, where extruded inks are rapidly cured and layered to create stacked structures^22–26^. Fabricating 3D conductive pathways based on direct extrusion printing techniques has been extensively studied^27–29^. Seol et al.^30^ employed a low-viscosity ink composed of silver nanoparticles (AgNPs), where conductive microstructure printing was facilitated by microwave heating. The reduced silver content in this ink led to high resistivity in the printed structures. Lewis et al.^31^ fabricated high-precision, interconnected silver microelectrodes using concentrated inks with higher viscosity AgNPs, which improved electrical conductivity. They use a variety of needles to print different sizes of silver thread. Lee et al.^32^ fabricated emulsified elastomeric ink containing AgNPs and carbon nanotubes, which gave the printing facility excellent tensile properties. The printing resolution was low due to the high viscosity of the composites. Overall, the structure of an extrusion print is influenced by the ink and the inner diameter of the needle. The structure’s resolution is generally greater than the inner diameter of the needle^33–36^. The print speed must match the ink volume to prevent structural breakage, which is related to the viscosity and curing speed of the ink^37–40^. Consequently, the constant single-needle internal diameter and ink curing speed make it difficult to increase print resolution and speed further.

Here, a fluid drawing micro-printing technique was proposed to overcome the limitations of extruded micro-printing technologies for 3D filamentary structures. During the printing process, the ink at the needle is in contact with the substrate, creating a high-precision 3D filamentary structure under tension during upward movement. This increases the printing speed while preparing a filamentary with a diameter smaller than the inner diameter of the needle. The solvents (29 Pa·s) in the inks are evaporated at 25–100 °C to increase the viscosity for drawing printing. 3D conductive structures of different wire widths were printed with the same needle at varying speeds, air pressure, and temperature. The conductive structure, obtained by thermal treatment, exhibits high conductivity and favorable bending properties. Prototypes for flexible electronics have been developed, including light-emitting diode (LED) arrays, thermal imaging displays, and multivibrator circuits. All these devices leverage freestanding 3D conductive pathways, underscoring the unique benefits of fabricating complex flexible circuits.

## Results and discussion

### Principle of fluid drawing printing technology

**Fig. 1:**
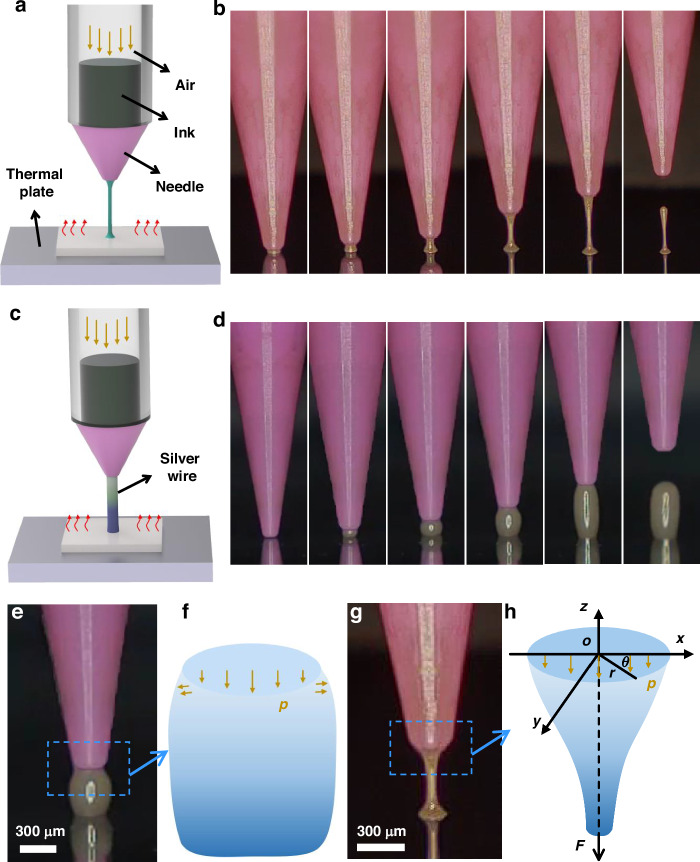
**Fluid drawing printing technique versus traditional extrusion printing technique.** **a** Schematic diagram of the fluid drawing printing technique method. **b** The stages of drawing printing. **c** Schematic diagram of the extrusion printing technique. **d** The stages of extrusion printing. **e** Localized enlarged view of extrusion printing. **f** Ink force analysis for extrusion printing. **g** Localized enlarged view of drawing printing. **h** Force analysis of the liquid bridge

Direct writing printing technology is promising for fabricating 3D conductive pathways. Extrusion-based methods, however, face challenges in achieving high precision and rapid production. This paper proposes a fluid drawing printing technique that facilitates the creation of 3D filamentary structures with wire diameters substantially smaller than the inner diameter of the needle and enables high-speed printing. Figure 1a shows a schematic diagram of the drawing printing method. The ink, stored in a syringe, is ejected to the substrate under air pressure. The substrate positioned on a thermal plate promotes the evaporation of the ink solvent, thereby increasing the viscosity of the ink and forming a paste. Lifting the needle stretches the paste between the needle and the substrate into filaments due to tension. These filaments solidify in a thermal field, forming a 3D filament structure.

In comparison, traditional printing of 3D filamentary structures relies mainly on extrusion, and the schematic diagram of the extrusion printing technique is shown in Fig. 1c. The ink is extruded onto the substrate under air pressure and then gradually solidifies due to the thermal field. The ink can withstand low tension, leading to structural breakage when printing too fast. The needle must ascend slowly to build up the ink on the solidified structure. The extrusion process under air pressure is depicted in Fig. 1d. As the needle moves upward, the height of the silver column increases, and the structure becomes thicker due to the air pressure, as illustrated in Fig. 1e, f. Throughout the printing process, the ink is only influenced by the internal pressure within the syringe.

Unlike the traditional extrusion printing technique, the proposed fluid drawing printing method utilizes controls for forming the structure through tension. Figure 1b illustrates the drawing printing stages for silver columns, where a tensile force affects the paste between the substrate and the needle as the needle ascends. As depicted in Fig. 1g, the paste forms a liquid bridge at the needle under tension^41^. The ink is transported under air pressure to the liquid bridge and forms a paste in the presence of a thermal field. The silver wire is drawn from the paste under tension and solidifies. Consequently, it becomes feasible to print a silver wire with a wire diameter smaller than the inner diameter of the needle. The liquid bridge during drawing printing technology affects the printed silver wire.

A force analysis of the liquid bridge is performed to determine the factors affecting it (Fig. 1h). A coordinate system is established at the upper end of the liquid bridge, with the central axis of symmetry as the *z*-axis downward as positive and *o* as the origin. According to the continuity equation of the fluid ^42^,

1 $$\frac{\partial {v}_{r}}{\partial r}+\frac{1}{r}\frac{\partial {v}_{\theta }}{\partial \theta }+\frac{\partial {v}_{z}}{\partial z}+\frac{{v}_{r}}{r}=0$$

where *r*, *θ*, and *z* are the cylindrical coordinates built on the liquid bridge; *v*_i_ is the velocity component of the mass motion in the liquid bridge structure. The positive stress tension per unit area of the liquid bridge can be calculated by substituting the boundary conditions of the liquid bridge into the continuity equation. The detailed calculation is mentioned in Equations S1–S10. The calculation determined the total tension in the liquid bridge cross-section to be,

2 $$F=2\mu s\frac{\partial {v}_{z}}{\partial z}$$

where *μ* is the material’s kinetic viscosity coefficient; *s* is the cross-sectional area. The above equation shows that the tensile tension *F* applied to the liquid bridge is proportional to the viscosity coefficient, cross-section, and velocity gradient. Tension stabilization is essential for maintaining the tensile quality of the liquid bridge.

### Fluid drawing printing formation analysis

Based on the abovementioned analysis, tension is primarily influenced by viscosity coefficient (*μ*) and velocity gradient. The viscosity of the ink and solvent evaporation governs the viscosity of the liquid bridge. Therefore, preparing inks and regulating their viscosity is crucial for drawing print. A high-viscosity ink formulation containing AgNPs-PVP with DMF as the primary solvent was developed for drawing printing technology. PVP is used to disperse the silver particles and increase the viscosity of the ink. AgNPs are encapsulated by PVP. The viscosity of the prepared ink was 29 Pa∙s, significantly higher than typical inkjet inks (2–30 mPa∙s)^43^. The initial ink tends to break easily, facilitating the separation of the needle from the structure after printing.

The solvent’s evaporation further increases the viscosity of the ink, thus resulting in the realization of a drawing printing. DMF is a soluble and volatile solvent that can quickly evaporate at high temperatures. Lower temperatures result in reduced solvent evaporation rates, thus leading to minimal changes in ink viscosity. On the contrary, as the temperature increases, the solvent evaporation rate increases, promoting the entanglement of the PVPs with each other and an increase in the interaction. The ink quickly forms a paste. As shown in Fig. S5, the viscosity of the ink increases gradually with increasing temperature. At 80 °C, the viscosity of the ink reaches 8.2 × 10^4^ mPa∙s, facilitating the fluid drawing printing.

**Fig. 2:**
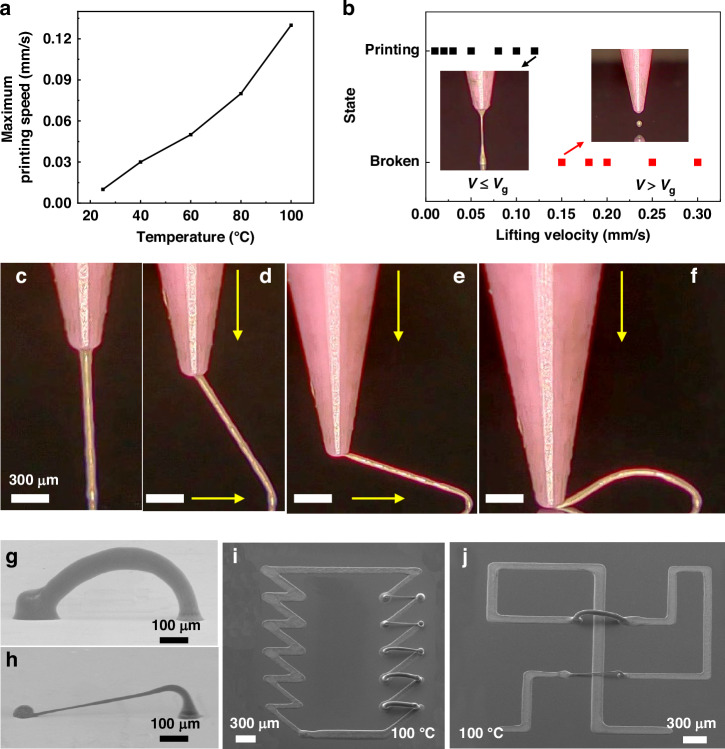
**Formation of draw prints**. **a** Relationship between temperature and maximum printing speed. **b** The state of the silver wire about the nozzle lifting velocity. Printed 90° (**c**), 45° (**d**), and 30° (**e**) silver wires. **f** The printing of the 3D filamentary structure. 3D filamentary structures were printed at speeds of 20 μm/s (**g**) and 80 μm/s (**h**). **i** SEM image of planes and 3D architectures. **j** SEM images of crossed and isolated circuits

The drawing printing was investigated by analyzing the ink printed and broken under different temperatures and lifting velocities, as illustrated in Figs. S6 and S7. When the solvent volatilizes less, the ink has too low a viscosity to form a liquid bridge and break. As the thermal plate temperature increases, the maximum speed for drawing prints gradually increases, as shown in Fig. 2a. At a constant speed, the silver wire’s stability and diameter increase as the temperature rises. This is because the liquid bridge becomes more viscous as the temperature increases and the velocity decreases. As shown in Fig. 2b, the effects of lifting velocity (*v*) on wire printing and wire breaking were determined when the temperature of the thermal plate was set at 100 °C. When the lifting velocity is below the threshold (*v*_g_), the silver wire remains attached to the needle (as shown in the black data points of Fig. 2b and the inset below). When lifting velocity exceeds the threshold, the needle separates from the silver wire (as shown in the red data points of Fig. 2b and the inset above).

The 3D filamentary structure was fabricated by moving a needle to draw the silver wire out and attach it to another point on the substrate. Figure 2c illustrates a vertical silver wire printed at an air pressure of 2.5 kPa and a speed of 50 μm/s. Figure 2d, e demonstrates that by moving the substrate to the right and the needle downward, the angles of the silver wire relative to the substrate are adjusted to 45° and 30°, respectively. The 3D filament structure is directly printed by lowering the needle to contact the substrate, as illustrated in Fig. 2f. The silver wire, printed at 20 μm/s, exhibited a wire width of 100 μm. This 3D filamentary structure has a pronounced curvature and a uniform diameter, as depicted in Fig. 2g. The silver 3D filamentary structure, printed at 80 μm/s, is illustrated in Fig. 2h. The diameter of the silver wire remains uniform along its length but thickens near the substrate. Since the diameter of the silver wire is smaller than the diameter of the nozzle. At the substrate position, the needle nozzle determines the diameter of the silver wire. During the lifting process, the silver wire becomes progressively thinner from the diameter of the nozzle.

To demonstrate the application of 3D filamentary structures in printed circuits, silver structures with 2D and 3D wire features can be created by precisely controlling printing speed and direction. Figure 2i and Movie S1 illustrate the planes and 3D architectures with varying wire widths. Parallel lines of varying widths are directly printed onto the plane, while 3D architectures are printed as an initial point to ensure closed connections. Figure 2j and Movie S2 show the crossing circuits created by successive printing of planes and 3D filament architectures. After printing the 2D pattern, the needle is gradually lifted from the substrate so that 3D filamentary structures can be printed directly. 2D wires were printed directly by moving the needle to a predetermined planar position. As a result, cross and isolated 3D architectures have been completed.

### 3D filamentary structure printing with different wire widths

**Fig. 3:**
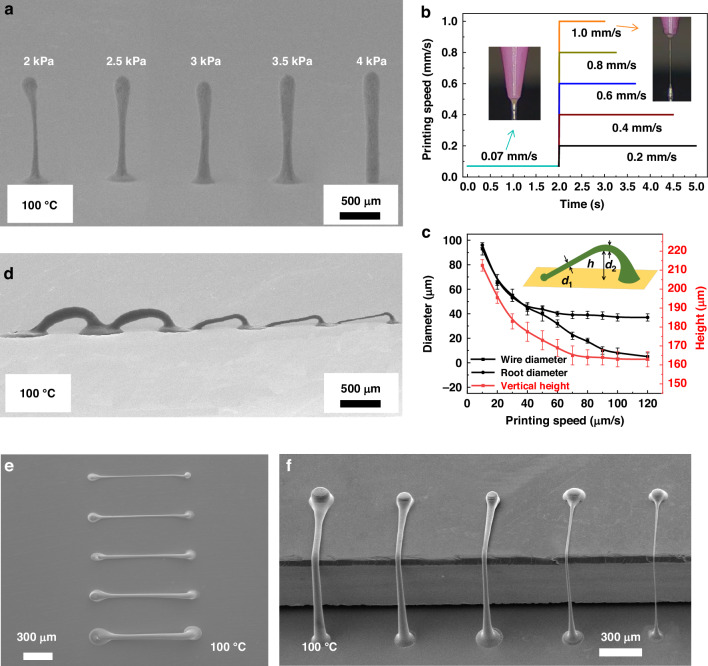
**Printing of 3D filamentary structures with different wire widths**. **a** Silver columns printed at different air pressures. **b** Variable speed printing silver wire construction. **c** The effect of various printing speeds on the height of 3D filamentary structures. **d** SEM images of successively printed silver structures with varying widths of wire. **e** SEM image reveals significant spanning 3D filamentary structure arrays with varying wire widths. **f** SEM image reveals a significant spanning 3D filamentary structure printed on a step

3D filamentary structures can be printed with different wire widths using the same needle, which has significant potential in circuit design. The wire width of the printed silver wire depends on temperature, air pressure, and print speed. The experiment above demonstrated that from room temperature to 100 °C, the diameter and stability of the silver wire increase with rising temperature. The slow rate of temperature increase, however, affects printing efficiency. Hence, the thermal plate temperature was maintained at 100 °C, with pressures ranging from 2 to 4 kPa and speeds ranging from 10 to 60 μm/s to print silver columns. Fig. S8 illustrates that the wire width of the silver column decreases as air pressure decreases and print speed increases. This occurs due to a decrease in air pressure, resulting in a reduction of deposited ink. An increase in printing speed leads to an increase in the speed gradient. These causes lead to a reduction in the diameter of the silver wire. The maximum wire width reaches 180 μm at an air pressure of 4 kPa and a printing speed of 10 μm/s. Figure 3a and Movie S3 display the silver columns printed within an air pressure range of 2–4 kPa and at a print speed of 60 μm/s. When the pressure falls below 3 kPa, the top diameter of the silver column is noticeably larger. Such a feature results from a reduction in liquid bridge curvature caused by the decreased wire width of the silver column. When the silver column separates from the needle, the ink accumulates on the silver column.

In addition, a variable speed printing process was developed, i.e., the printing process is increased by two stages, as shown in Fig. 3b. Low-velocity atmospheric pressure (0.07 mm/s and 3.5 kPa were used here) at stage one increases the ink at the liquid bridge, which is used to accelerate solvent volatilization. Increase print speed and reduce air pressure (2.5 kPa) in stage two for fast printing. The maximum print speed is 1.0 mm/s, and the diameter of the silver wire is 4.39 ± 1.37 μm. This is due to the increased viscosity of the bridge at low speeds, which allows the bridge to withstand greater tensile forces. The silver wires printed at different speeds are shown in Fig. S9. As the speed increases, the diameter of the silver wire decreases slightly, which is the same as the uniform speed printing.

3D filamentary structures with different line widths are printed with the traction of a needle. Figure 3c illustrates the relationship between printing speeds, wire diameter (*d*_1_), root wire diameter (*d*_2_), and vertical height (*h*) at an air pressure of 2.5 kPa. The wire diameter decreases gradually with increased printing speed. The diameter measures 5.52 ± 1.41 μm at a printing speed of 120 μm/s. The root diameter remains nearly constant for printing speeds exceeding 60 μm/s. This can be attributed to the correlation between the root diameter and the inner diameter of the nozzle. The overall vertical height of the structure exhibits minimal variation because of the slight fluctuations in the root diameter. The minimum vertical height measures approximately 37 µm. Figure 3d and Movie S4 show 3D filamentary structures with different wire widths printed continuously using different air pressures and speeds.

3D filamentary structures with varying wire widths facilitate the preparation of large-span prints on flat surfaces and steps. Figure 3e and Movie S5 show 3D filamentary structure arrays with different wire widths and large spans. Silver wire with a high aspect ratio featuring a wire width of 10 µm and a length of 1.2 mm (aspect ratio of 120) is pre-printed at an air pressure of 2 kPa and a speed of 70 µm/s. The nozzle is then adjusted to anchor the second point of the silver wire to the substrate, enabling 3D architecture. Figure 3e shows a gradual increase in linewidth from top to bottom printing by decreasing the writing speed and increasing the air pressure, which reflects the 3D filamentary structure’s linewidth modulation. The diameter of the contact point between the silver wire and the substrate is about 60-200 μm, determined by the contact between the liquid bridge ink and the substrate. Figure 3f shows bridges with different wire widths printed on a 600 μm step. The needle remained in contact with the silver wire throughout the printing process without breaking.

### Performance analysis of freestanding 3D filamentary structures

**Fig. 4:**
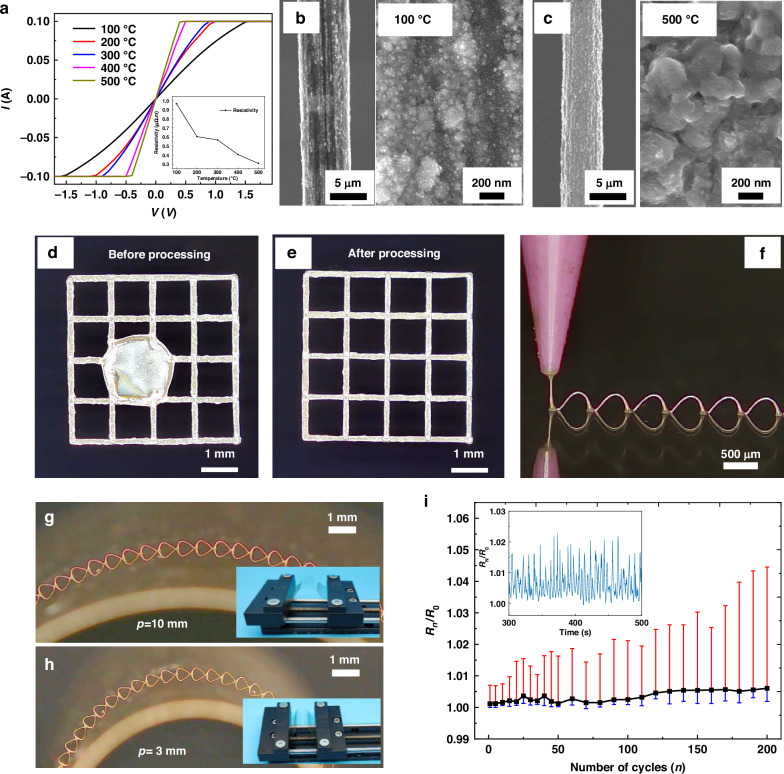
**Performance test of thermally treated 3D filamentary architectures**. **a** The relationship between voltage and current at different thermal treatment temperatures. The inset shows the relationship between thermal treatment temperature and silver wire resistivity. SEM images show the silver wires after thermal treatment at 100 °C (**b**) and 500 °C (**c**). **d** The pattern without thermal treatment is broken to form a large circle. **e** The thermally treated pattern is preserved with no deformation. **f** Continuously printed architecture of silver wires. Pictures of silver wires at 10 mm (**g**) and 3 mm (**h**) radius of curvature. **i** The resistance change (*R*_n_) is normalized after *n* cycles to the initial resistance (*R*_0_) before cyclic testing. The inset shows the resistance change curve

Printed 3D filamentary structures have poor electrical conductivity due to solvent and PVP. In order to realize the application of printed filamentary structures in circuits, the conductivity after thermal treatment needs to be investigated. As shown in Fig. 4a, silver wires’ current-voltage (*I*–*V*) characteristics with a wire width of approximately 5 μm have been evaluated after thermal treatment at various temperatures (100 °C to 500 °C). The inset of Fig. 4a illustrates a sharp decrease in the resistivity of the architecture as the temperature increases. The resistivity of the silver wire is 5.6 × 10^-7^ Ω∙m after thermal treatment at 300 °C for 30 min, which closely corresponds to the resistance value of silver (10^-8^ Ω∙m). The resistivity decreases to 2.5 × 10^-7^ Ω∙m after thermal treatment at 500 °C for 30 min. This demonstrates that it is possible to prepare freestanding 3D filamentary structures with varying resistances by controlling the thermal treatment temperature and the diameter and length of the printing filament. Figure 4b, c displays the scanning electron microscope (SEM) images of the 3D silver wires after thermal treatment at various temperatures. Thermal treatment at 500 °C removes the PVP within the 3D filamentary structure, exposing the AgNPs entirely. The particles within the structure become more densely packed, with the particle size increasing from 20 nm to 200 nm as the thermal treatment temperature rises from 100 °C to 500 °C.

The removal of PVP from the ink and the reduction of functional groups during thermal treatment significantly enhanced the hydrophobicity of the structure^29^. Two 4 × 4 grid patterns with a side length of 1 mm were printed on a plane to verify the thermal treatment’s effect on printed structures’ stability. One of the patterns was thermally treated at 150 °C. The deionized water droplets precisely control to drop onto the two patterns respectively. Water stability tests were performed on the structures without and after thermal treatment, as shown in Fig. 4d, e. The pattern without thermal treatment was broken to form a large coffee ring. After thermal treatment, the patterns remain undistorted, demonstrating that the thermally treated structures have improved water stability.

Cyclic bending tests were conducted on the printed continuous silver wire to assess the stability of the freestanding 3D interconnect. Polyimide (PI) film, which is widely used in flexible electronics due to its stable performance and high-temperature resistance, served as the substrate. Figure 4f depicts the drawing printing of a continuous 3D filament on the PI film. A cyclic bending test was performed on a thermally treated silver wire at 150 °C. The insets of Fig. 4g, h display images of the bending device. Figure 4g, h illustrates that the structure of the 3D silver wire remains unchanged during bending with radii of curvature of 10 mm and 3 mm, respectively. Figure 4i demonstrates the resistance variations over 200 test cycles, with the inset highlighting the resistance changes during bending. The resistance of the continuous 3D bridge (Fig. 4f) increases with the number of bends; the maximum difference in resistance is less than 5%. This continuous freestanding 3D interconnect retains its shape and conductivity even under repeated bending cycles, indicating excellent flexibility and durability.

### Printed freestanding 3D conductive structures for flexible electronics

**Fig. 5:**
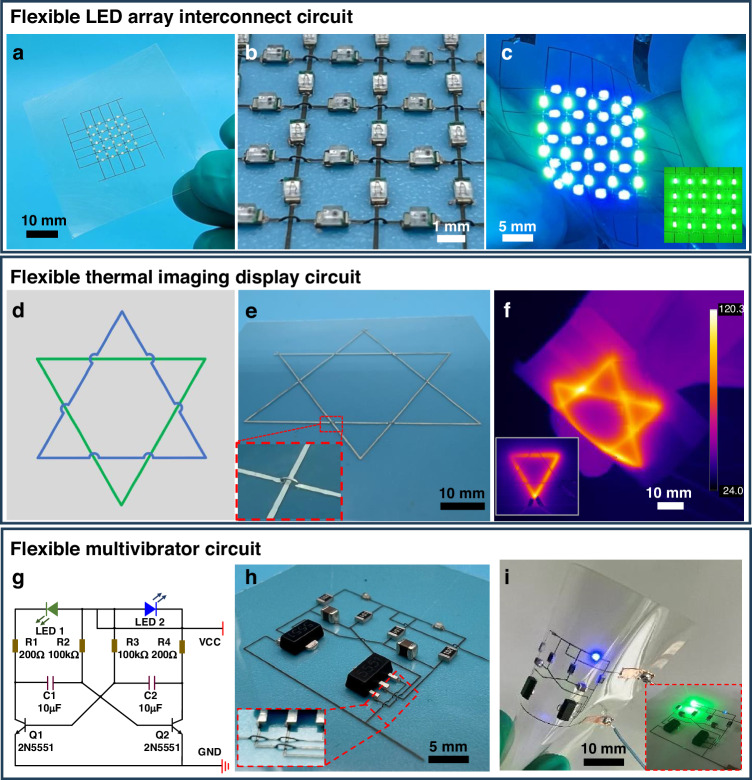
**Printed freestanding 3D conductive structures for flexible electronics**. **a** Optical image of the prepared LED array. **b** Enlarged view of the LED array. **c** Pictures of LED arrays operating in a bent state. The inset shows a working diagram of a longitudinal LED array. **d** A schematic diagram of the flexible thermal imaging display devices. **e** Photos of the prepared flexible thermal imaging display devices. The inset shows an enlarged view of the freestanding 3D interconnect. **f** The flexible thermal imaging displays in a bending state. The inset is a thermal image of a single inverted triangle. **g** Schematic diagram of multivibrator circuit. **h** Images of the prepared multivibrator circuit. The inset is an enlargement of a portion of the freestanding 3D interconnect. **i** Test image of the multivibrator circuit. The inset shows a schematic of the multivibrator circuit in the planar state

Fluid drawing printing, suitable for various material substrates, offers multiple applications in flexible electronics manufacturing. This technology enables the fabricating of interconnects horizontally and vertically onto the surface of flexible films, thereby minimizing circuit complexity. Flexible LED array circuits can be used in displays and lighting. The increase in the number of LEDs increases the complexity of the circuits. As demonstrated in Fig. 5a, the LED array fabricated on the PI film utilizes a printed circuit. Figure 5b presents an enlarged view of the LED circuit, illustrating the establishment of freestanding 3D interconnect at the intersections of horizontal and vertical wires to prevent short circuits. The LEDs can be independently operated in both the transversal and longitudinal directions by applying a bias voltage to the circuit. Figure 5c and Movie S6 show the operation of the LED array circuit over bending tests. The illustration in Fig. 5c shows a picture of the independent longitudinal LEDs in operation. The n×n horizontal and vertical LED arrays comprise two independent circuits. Traditional 2D interconnects require a cross-arrangement that can only be achieved through two-layer printing and through-hole connections. In contrast, with 3D filamentary structures, the interconnect needs only 2n lines in the plane, significantly reducing the interconnections and the required space.

A flexible thermal imaging display device was fabricated as a demonstration based on freestanding 3D conductive structures. Two separate triangles (blue and green) were arranged as depicted in Fig. 5d. The wires at the intersection of the two triangles were printed with freestanding 3D conductive structures to prevent short circuits. The positive graph (in blue) and the reverse graph (in green) can operate independently. Mica sheets can withstand high temperatures over 1000 °C. The mica sheet is used as a flexible substrate. After thermal treatment at 350 °C, the thermal imaging display device is shown in Fig. 5e. The planar and 3D filamentary structure is well maintained. Such planar and 3D interconnects hold up well, ensuring the independent operation of the two triangular circuits. Figure 5f and Movie S7 show the circuit’s operation under bending. The device maintains its structural integrity after multiple bends in different directions. When two triangles run simultaneously, the temperature at the interconnect point is significantly higher than elsewhere. This is due to the superposition of the temperatures of the upper and lower leads. The fact that the two triangles can operate independently without interference illustrates the isolation of the interconnect. This experiment demonstrated the stability of the fabricated flexible thermal imaging device with freestanding 3D interconnects.

The application of freestanding 3D conductive structures in complex circuits with multiple devices is validated. Printing on PI film to fabricate a multivibrator circuit was demonstrated. As illustrated in Fig. 5g, the multivibrator is constructed using two NPN (Negative-Positive-Negative) triodes and capacitor charging and discharging. One of the two triodes is conducting while the other triode is cut off. The circuit comprises two 2N5551 triodes, two 10 μF capacitors, two 200 Ω resistors, two 100 kΩ resistors, and two LEDs. Figure 5h describes images of the assembled circuit and the partially amplified 3D conductive structures. Since the multivibrator circuit has symmetry and cross circuits exist, the connecting circuits are simplified by printing a 3D conductive structure. As shown in Fig. 5i and Movie S8, when the circuit is connected to a 5 V power supply, the output voltage alternates due to capacitor charging and discharging, and the two LEDs flash alternately. The circuit remains undamaged and maintains its blinking function after multiple bends. This result demonstrates the stability of printed silver wires and interconnects for circuit applications. Three circuits prepared on a flexible substrate reduced circuit complexity by drawing printing freestanding 3D conductive structures. The strength of the printed architecture allows for stable operation under bending conditions.

According to the demonstration above, we have developed a fluid drawing method to print 3D conductive structures. The ink remains fluid before printing and solidifies afterward, creating an extreme change in viscosity. The liquid bridge structure is formed by tension during the printing process and realizes printing at different speeds and precision with a single-needle action. The state of the ink changes is mainly influenced by the original viscosity of the ink, the type of solvent, and the ambient temperature. Increased ink viscosity increases print speed, as shown in Table S3 of the supporting materials. High ink viscosity, however, can affect the print resolution. This paper aims to ensure the accuracy of the silver line while achieving fast printing. Variable speed printing was tested up to 1 mm/s, comparable to current direct printing speeds but with lower viscosity and approximately 4 µm wire diameter. To increase print speed and reduce issues about pores and cracks in the silver wires, adding other polymers or increasing hydrogen bonding can be considered for further optimization. The viscosity of the ink can be increased by combining various materials without reducing the silver content, thus mitigating the loss of electrical conductivity and the effect of solvent evaporation. Meanwhile, further improvements in annealing, such as gradient heating, can be implemented to minimize the problem of porosity caused by fast temperature change.

Silver paste accumulates at the nozzle during the drawing printing process, making it difficult to separate the nozzle from the silver wire. Consequently, this forms a contact point thicker than the linewidth, affecting printing precision (Figs. 2h, 3a, and 3f). Further efforts include analysis to mitigate the impact of thickened touchpoints. During the experiments, it was observed that utilizing the substrate as a heat source leads to decreased thermal radiation performance with increasing height, which affects the printing performance. The position of the thermal field can be designed and optimized for future studies to achieve omnidirectional and multi-angle thermal effects and improve printing performance. This approach efficiently realizes high-resolution, multi-scale, 3D interconnect printed structures, which is essential for further circuits. Overall our work is a good example illustrating the importance of viscosity alteration in the additive manufacturing process. It could encourage further exploration into ink development, including changes in properties. More material systems could be adapted. These findings could serve as valuable insights for future additive manufacturing.

## Conclusion

A fluid drawing printing technique has been proposed in which AgNP paste is drawn rather than extruded. During the rapid printing process, the silver wire was refined without breaking, enabling the fabrication of high-precision (4 μm) and high-speed (1 mm/s) 3D filamentary structures. The force analysis of the liquid bridge during the drawing printing process has been performed to determine the relationship between the tension during the printing process and the viscosity and speed. High-viscosity inks consisting of AgNPs, PVP, and DMF have been prepared. Silver wire fabrication was realized by increasing the temperature (from ambient temperature to 100 °C) to adjust the viscosity and validate the application of printed 3D architectures in circuits. 3D filamentary structures with different wire widths were prepared using a single needle in our method completed. The effect of varying air pressures and speeds on the wire widths has been tested. Printing 5–180 μm line widths was achieved with a 50 μm needle at 2–4 kPa and 10–120 μm/s printing speeds. After thermal treatment at 500 °C, the size of the silver particles of the interconnect increases to 200 nm, and the resistivity decreases to 2.5 × 10^-7^ Ω∙m. This technique has been applied to various flexible circuits, including LED arrays, thermal imaging displays, and multivibrator circuits, by printing planes and freestanding 3D conductive structures to simplify circuit complexity. The proposed drawing printing method by varying viscosity offers guidance and practical prerequisites for optimizing 3D interconnect. It holds significant potential for rapid and precise flexible circuit manufacturing.

## Methods

### Printing inks containing PVP-AgNPs were prepared

A solution was prepared by adding 1 g of PVP to 3 g of DMF and 1 g of diethylene glycol monomethyl ether (DEM). The solution was obtained by stirring the PVP and the solvent with a magnetic stirrer for 20 min until thoroughly mixed. The solution was supplemented with 10 g of 20 nm silver powder. The solution was subjected to 2 h of ultrasonic shaking for uniform dispersion. Incorporate 0.03 g of propanetriol to adjust the viscosity and introduce 0.01 g of hydroxymethylcellulose as a bonding agent. The solution was subjected to magnetic stirring for 2 h to ensure uniform mixing. A high-viscosity drawing printing ink containing 6.5% PVP was acquired.

### The experimental setup for PVP-AgNps ink printing was established

The system incorporates a moving stage (UKSA200, Zolix, China) that enables three degrees of freedom movement. A 1 mL syringe was used, and a needle (50 μm, Musashi, Japan) with an inner diameter of 50 μm was installed. Air is supplied to the upper end of the syringe through the dispenser (Super ΣCMII-V2, Musashi, Japan), connected to a pressure-reducing valve (RP-0.5-2, Fujikura, Japan) via an air hose. The thermal plate is fabricated using an aluminum alloy, and the heating wire is controlled by a thermostat (E5CC-800, Omron, Japan). The camera performs real-time observation of the writing process (BC1000, Bocheng, China).

### The microstructure of silver was characterized

The properties of inks were evaluated utilizing an advanced rheometer (AR2000ex, TA Instruments, USA). The printed structures undergo thermal treatment in a muffle furnace (LHL-14CMA-3, Haheng, China). The change in mass during the sintering of silver ink is measured using a thermogravimetric analyzer (TGA/SDTA851e, Mettler Toledo, Switzerland). Field emission scanning electron microscopy (JSM-7610F Plus, JEOL, Japan) is utilized to characterize the surface morphology of the structure.

### The performance of printed circuits was tested

The silver wires are routed through a parametric tester (4200, Keithley Keithley, USA) to examine their *I*–*V* curve. The maximum current density of the silver wire is measured using a precision source/measure unit (B2900A, Agilent, USA). The Young’s modulus and hardness were tested using a nanoindentation tester (100BA-1C, MTS, USA). The resistance change of the silver wires in real-time during a bending test is measured using a digital multimeter (34410A, Agilent, USA). A DC-regulated power (DP831, Rigol, China) supply and an adjustable DC power (HSPY-3010, Hanshengpuyuan, China) supply are employed to apply voltage bias to the flexible circuit. The heat generation of the silver wire is recorded using an infrared camera (Ti480 Pro, Fluke, USA).

## Supplementary information

Supplementary pictures and calculations for liquid bridges

Planar and 3D interconnects of different line widths

Printing crossover and isolation circuits with different line widths

Printing silver columns at varying air pressures

Prints continuous 3D filament structures with different line widths

Printing spanning 3D interconnects with different line widths

Flexible crossover LED circuit experiment

Flexible thermal imaging display experiment

Flexible multivibrator circuit experiment

## Data Availability

The data supporting this study’s findings are available from the corresponding author upon reasonable request.

## References

[1] Jung, D. et al. Highly conductive and elastic nanomembrane for skin electronics. *Science* **373**, 1022–1026 (2021). 34446604 10.1126/science.abh4357

[2] Liu, H. et al. Flexible electronics based on organic semiconductors: from patterned assembly to integrated applications. *Small* **19**, e2206938 (2023). 36642796 10.1002/smll.202206938

[3] Huang, Q. et al. Printing conductive nanomaterials for flexible and stretchable electronics: a review of materials, processes, and applications. *Adv. Mater. Technol.* **4**, 1800543 (2019).

[4] Nayak, L. et al. A review on inkjet printing of nanoparticle inks for flexible electronics. *J. Mater. Chem. C.* **7**, 8771–8795 (2019).

[5] Khan, Y. et al. A new frontier of printed electronics: flexible hybrid electronics. *Adv. Mater.* **32**, e1905279 (2020). 31742812 10.1002/adma.201905279

[6] Mitra, K. Y. et al. Fully inkjet-printed thin-film transistor array manufactured on paper substrate for cheap electronic applications. Adv. Electron. *Adv. Electron. Mater.* **3**, 1700275 (2017).

[7] Kopola, P. et al. Gravure printed flexible organic photovoltaic modules. *Sol. Energy Mater. Sol. Cells* **95**, 1344–1347 (2011).

[8] He, P. et al. Screen-printing of a highly conductive graphene ink for flexible printed electronics. *ACS Appl Mater. Interfaces* **11**, 32225–32234 (2019). 31390171 10.1021/acsami.9b04589

[9] Li, D. et al. Printable transparent conductive films for flexible electronics. *Adv. Mater.* **30**, 1704738 (2018). 10.1002/adma.201704738 29319214

[10] Cheng, T. et al. Inkjet-printed high-performance flexible micro-supercapacitors with porous nanofiber-like electrode structures. *Small*. **15**, 1901830 (2019). 10.1002/smll.201901830 31293068

[11] Chen, Z. et al. Systematic investigation of novel, controlled low-temperature sintering processes for inkjet printed silver nanoparticle ink. *Small*. **20**, 2306865 (2023). 10.1002/smll.202306865 38126669

[12] Kim, Y. Y. et al. Roll-to-roll gravure-printed flexible perovskite solar cells using eco-friendly antisolvent bathing with wide processing window. *Nat. Commun.* **11**, 5146 (2020). 33051454 10.1038/s41467-020-18940-5 PMC7555830

[13] Liang, J. et al. A water‐based silver‐nanowire screen‐print ink for the fabrication of stretchable conductors and wearable thin‐film transistors. *Adv. Mater.* **28**, 5986–5996 (2016). 27159406 10.1002/adma.201600772

[14] Kamyshny, A. et al. Conductive nanomaterials for 2D and 3D printed flexible electronics. *Chem. Soc. Rev.* **48**, 1712–1740 (2019). 30569917 10.1039/c8cs00738a

[15] Phung, T. H. et al. Hybrid fabrication of LED matrix display on multilayer flexible printed circuit board. *Flex. Print. Electron*. **6**, 024001 (2021).

[16] Wang, M. et al. Stencil printing of liquid metal upon electrospun nanofibers enables high-performance flexible electronics. *ACS Nano* **15**, 19364–19376 (2021). 10.1021/acsnano.1c05762 34783541

[17] Dahiya, A. S. et al. Printed interconnects for heterogeneous systems integration on flexible substrates. *Adv. Mater. Technol.* **10**, 2401213 (2024).

[18] Wei, H. et al. Direct 3D printing of hybrid nanofiber-based nanocomposites for highly conductive and shape memory applications. *ACS Appl. Mater. Interfaces* **11**, 24523–24532 (2019). 31187627 10.1021/acsami.9b04245

[19] Zhou, L. Y. et al. A review of 3D printing technologies for soft polymer materials. *Adv. Funct. Mater.* **30**, 2000187 (2020).

[20] Ren, P. et al. Direct Fabrication of VIA interconnects by electrohydrodynamic printing for multi-layer 3D flexible and stretchable electronics. Adv. *Mater. Technol.* **6**, 2100280 (2021).

[21] Wang, Y. et al. Multilayer flexible electronics: manufacturing approaches and applications. *Mater. Today Phys*. **23**, 100647 (2022).

[22] Yuk, H. et al. 3D printing of conducting polymers. *Nat. Commun.* **11**, 1604 (2020). 32231216 10.1038/s41467-020-15316-7 PMC7105462

[23] Hou, Z. et al. Direct ink writing of materials for electronics-related applications: a mini review. *Front. Mater*. **8**, 647229 (2021).

[24] Saadi, M. A. S. R. et al. Direct ink writing: a 3D printing technology for diverse materials. *Adv. Mater.* **34**, 2108855 (2022). 10.1002/adma.202108855 35246886

[25] Tang, M. et al. Advanced supramolecular design for direct ink writing of soft materials. *Chem. Soc. Rev.* **52**, 1614–1649 (2023). 36779285 10.1039/d2cs01011a

[26] Jo, Y. et al. 3D-printable, highly conductive hybrid composites employing chemically-reinforced, complex dimensional fillers and thermoplastic triblock copolymers. *Nanoscale* **9**, 5072–5084 (2017). 28181617 10.1039/c6nr09610g

[27] Hu, J. et al. Meniscus-confined three-dimensional electrodeposition for direct writing of wire bonds. *Science* **329**, 313–316 (2010). 20647464 10.1126/science.1190496

[28] Skylar-Scott, M. A. et al. Laser-assisted direct ink writing of planar and 3D metal architectures. *Proc. Natl Acad. Sci. USA* **113**, 6137–6142 (2016). 27185932 10.1073/pnas.1525131113 PMC4896727

[29] Kim, J. H. et al. Three-dimensional printing of highly conductive carbon nanotube microarchitectures with fluid ink. *ACS Nano* **10**, 8879–8887 (2016). 27564233 10.1021/acsnano.6b04771

[30] Lee, S. et al. Three-dimensional printing of silver microarchitectures using newtonian nanoparticle inks. *ACS Appl. Mater. Interfaces* **9**, 18918–18924 (2017). 28541035 10.1021/acsami.7b02581

[31] Ahn, B. Y. et al. Omnidirectional printing of flexible, stretchable, and spanning silver microelectrodes. *Science* **323**, 1590–1593 (2009). 19213878 10.1126/science.1168375

[32] Lee, B. et al. Omnidirectional printing of elastic conductors for three-dimensional stretchable electronics. *Nat. Electron.* **6**, 307–318 (2023).

[33] Gannarapu, A. et al. Freeze-printing of liquid metal alloys for manufacturing of 3D, conductive, and flexible networks. *Adv. Mater. Technol.* **1**, 1600047 (2016).

[34] Sun, P. et al. Directly printed interconnection wires between layers for 3D integrated stretchable electronics. *Adv. Mater. Technol.* **7**, 2200302 (2022).

[35] Kim, F. et al. Direct ink writing of three-dimensional thermoelectric microarchitectures. *Nat. Electron.* **4**, 579–587 (2021).

[36] Robertson, I. D. et al. Rapid energy-efficient manufacturing of polymers and composites via frontal polymerization. *Nature* **557**, 223–227 (2018). 29743687 10.1038/s41586-018-0054-x

[37] Park, Y. G. et al. Three-dimensional, high-resolution printing of carbon nanotube/liquid metal composites with mechanical and electrical reinforcement. *Nano Lett.* **19**, 4866–4872 (2019). 30983359 10.1021/acs.nanolett.9b00150

[38] Kim, J. T. et al. Three-dimensional writing of conducting polymer nanowire arrays by meniscus-guided polymerization. *Adv. Mater.* **23**, 1968–1970 (2011). 21523833 10.1002/adma.201004528

[39] Zhou, N. et al. Gigahertz electromagnetic structures via direct ink writing for radio-frequency oscillator and transmitter applications. *Adv. Mater.* **29**, 1605198 (2017). 10.1002/adma.201605198 28198059

[40] Park, Y.-G. et al. High-resolution, reconfigurable printing of liquid metals with three-dimensional structures. *Sci. Adv*. **5**, eaaw2844 (2019). 31245538 10.1126/sciadv.aaw2844 PMC6588379

[41] Xu, L. et al. Improved liver intravital microscopic imaging using a film-assisted stabilization method. *ACS Sens.* **9**, 5284–5292 (2024). 39228132 10.1021/acssensors.4c01464

[42] Luzi, G. et al. Study of the effects of inner pressure and surface tension on the fibre drawing process with the aid of an analytical asymptotic fibre drawing model and the numerical solution of the full N.–St. equations. *Arch. Appl. Mech.* **83**, 1607–1636 (2013).

[43] Guo, Y. et al. Inkjet and inkjet-based 3D printing: connecting fluid properties and printing performance. *Rapid Prototyp. J.* **23**, 562–576 (2017).

