# Supplementary material for: Fluid drawing printing 3D conductive structures for flexible circuit manufacturing
Source: Microsyst Nanoeng. 2025 May 12;11:81. doi: 10.1038/s41378-025-00936-0 (PMC12069710; doi:10.1038/s41378-025-00936-0)
Supplement: Supplementary file 1 — Supplementary pictures and calculations for liquid bridges [file 41378_2025_936_MOESM1_ESM.docx]

Supporting Information

**Fluid drawing printing 3D conductive structures for flexible circuit manufacturing**

Yikang Li^1^, Dazhi Wang^1, 2, 3, 4 *^, Yiwen Feng^1, 3^, Xiangji Chen^1^, Xu Chen^1^, Chang Liu^1^, Yanteng Li^1^, Liujia Suo^1^, Ran Zhang^1, 3^, Xiaopeng Zhang^5^, Ben Liu^1^, Fengshu Wang^1^, Shiwen Liang^4^, Lingjie Kong^4^, Qiang Fu^6^, Tongqun Ren^1^ and Tiesheng Wang^1, 3 *^

^1^ *Key Laboratory for Micro/Nano Technology and System of Liaoning Province, Dalian University of Technology, Dalian, 116024, China*

^2^ *State Key Laboratory of High-performance Precision Manufacturing, Dalian University of Technology, Dalian, 116024, China*

^3^ *Liaoning Huanghai Laboratory, Dalian, 116024, China*

^4^ *Ningbo Institute of Dalian University of Technology, Ningbo, 315000, China*

^5^*State Key Laboratory of Structural Analysis for Industrial Equipment, Dalian University of Technology, Dalian 116024, China.*

^6^*Ningbo Sunny Opotech Co., Ltd, Ningbo, 315400, China*

^*^*E-mail addresses: d.wang@dlut.edu.cn, wangts@dlut.edu.cn.*

A coordinate system is established at the superior end of the liquid bridge, with the central axis of symmetry as the *z*-axis, downward as positive, and *o* as the origin. The force analysis of the stress tensor component of the liquid bridge using cylindrical coordinates is as follows.

$\begin{matrix} \sigma_{rr}=-p+2\mu\frac{\partial v_{r}}{\partial r} \\ \sigma_{\theta\theta}=-p+2\mu\left[\frac{\partial}{\partial\theta}\left(\frac{v_{\theta}}{r}\right)+\frac{v_{r}}{r}\right] \\ \sigma_{zz}=-p+2\mu\frac{\partial v_{z}}{\partial z} \\ \sigma_{r\theta}=\mu\left[\frac{1}{r}\frac{\partial v_{r}}{\partial\theta}+r\frac{\partial}{\partial r}\left(\frac{v_{\theta}}{r}\right)\right] \\ \sigma_{z\theta}=\mu\left[\frac{1}{r}\frac{\partial v_{z}}{\partial\theta}+r\frac{\partial}{\partial z}\left(\frac{v_{\theta}}{r}\right)\right] \\ \sigma_{rz}=\mu\left[\frac{\partial v_{r}}{\partial z}+\frac{\partial v_{z}}{\partial r}\right] \end{matrix}$ (S1)

*σ*_ij_ is the stress tensor component; *r*, *θ*, and *z* are the cylindrical coordinates built on the liquid bridge; *v*_i_ is the velocity component of the mass motion in the liquid bridge structure; *μ* is the kinetic viscosity coefficient of the material; and *p* is the pressure inside the liquid bridge. According to the Navier-Stokes equation for the change of viscosity,

 (S2)

***v*** is the fluid vector; *t* is time; and *ρ* is the liquid density. This equation is derived for the case where the viscosity is constant. *μ* varies with time and position; the continuity equation is,

$\frac{\partial v_{r}}{\partial r}+\frac{1}{r}\frac{\partial v_{\theta}}{\partial\theta}+\frac{\partial v_{z}}{\partial z}+\frac{v_{r}}{r}=0$ (S3)

the liquid bridge is an axisymmetric structure with motion only in the *r* and *z* directions, *v_θ_*＝0. From the continuity equation multiplied by *r* on both sides of the equation,

$r\frac{\partial v_{r}}{\partial r}+v_{r}=-\frac{\partial v_{z}}{\partial z}r$ (S4)

integrate both sides concerning *r*,

$v_{r}=-\frac{1}{2}\frac{\partial v_{z}}{\partial z}r+\frac{C}{r}$ (S5)

where *C* is the constant of integration, when *r* = 0, *v*_r_＝0, so *C* = 0.

$v_{r}=-\frac{1}{2}\frac{\partial v_{z}}{\partial z}r$ (S6)

The viscous stress exists only when the fluid is in motion, and when the tension is removed, the viscous stress tensor is 0. Still, its internal pressure *p* is unchanged, so the tensile tension is independent of the internal pressure.

$\begin{matrix} \sigma'_{rr}=2\mu\frac{\partial v_{r}}{\partial r} \\ \sigma'_{\theta\theta}=2\mu\frac{V_{r}}{r} \\ \sigma'_{zz}=2\mu\frac{\partial v_{z}}{\partial z} \\ \sigma'_{r\theta}=0 \\ \sigma'_{z\theta}=0 \\ \sigma'_{rz}=\mu\frac{\partial v_{r}}{\partial z} \end{matrix}$ (S7)

The cosine of the direction of the cross-section is (*l m n*)＝(0 0 1). The stress per unit area in this section is ***f***,

 (S8)

The positive stress *N* per unit area is

$N=f_{r}l+f_{\theta}m+f_{z}n=2\mu\frac{\partial v_{z}}{\partial z}$ (S9)

Thus, the calculation of the total tension in the liquid bridge cross-section is publicized as

$F=\int_{s} Nds=\int_{s} 2\eta\frac{\partial v_{z}}{\partial z}rd\theta dr=2\mu s\frac{\partial v_{z}}{\partial z}$ (S10)

where *s* is the cross-sectional area.

The ink rheological properties with different polyvinylpyrrolidone (PVP) contents were tested. The increase in PVP content leads to an increase in ink viscosity. A significant shear thinning was observed (**Fig. S1**a) due to weakened intermolecular forces under shear stress. **Fig. S1**b illustrates the relationship between the storage modulus (G') and the loss modulus (G") of the prepared inks and shear stress. The storage modulus exceeds the loss modulus when the PVP content is low. The modulus decreases rapidly with increasing shear stress due to breaking molecular chains in the ink. The modulus is almost unaltered, with increasing shear stress and PVP content.

The SEM image of the silver ink before thermal treatment is shown in **Fig. S2**, which shows that the AgNPs are uniformly distributed and there is polymer between the particles. The size of silver particles is mainly ~20 nm, and a fraction of ~100 nm particles exists. To analyze the morphology of AgNPs was characterized using TEM, as shown in **Fig. S3**a. The silver particles are predominantly regularly rounded, showing well-defined edges and contours. This provides a view of the atomic arrangement within the silver particles, as well as the orientation and spacing of the lattice stripes, as shown in **Fig. S3**b.

The AgNP particle density in the ink was tested. Dilute 0.004 mL of ink with 350 mL of deionized water until translucent before TEM testing. The dilution with 10 μL was placed on a copper grid to dry and tested. The number of particles in the 2.9 μm×4.4 μm area is 61, as shown in **Fig. S3**a. The volume of diluent in this region is approximately 13.7 pL. The AgNP particle density of the ink was calculated to be approximately 390×10^15^ /mL. The size and intensity of silver particles in ink were tested using a granulometer, as shown in **Fig. S4**. The particle size was mainly 10-21 nm, with an intensity of 88.4 %. A small number of 100-200 nm particles were present with an intensity of 11.6 %.

Multiple silver wires were printed, and four samples were tested using SEM, with three areas selected for each sample and two randomly selected points tested to calculate the maximum print tolerance of the printed silver wires, as shown in **Fig. S10** and **Table S1**. The tolerance is calculated as the maximum limit size minus the minimum limit size. The maximum tolerance of the silver wire is calculated to be 1.41.

The weight changes of silver inks with different PVP contents have been detected using a thermogravimetric analyzer (TGA) using the same heating procedure. The heating process involves raising the temperature from 25 °C to 500 °C at a rate of 10 °C/min and then holding it at 500 °C for 30 minutes. **Fig. S11** depicts the variation of ink weight with temperature. Both inks experience a significant weight reduction when the temperature is below 200 °C. The weight loss of the ink during this period is attributed to solvent volatilization. Both inks showed comparable weight loss durations. Inks with higher PVP content have more significant weight loss. This is due to using the same solvents and the higher solvent content in the ink with the higher PVP content. Decomposition of the PVP in the ink occurs when the heating temperature reaches 400 °C. The weight of the printed PVP-AgNps ink decreased from 59.2% to 52.5%. The results demonstrate that the printed structure comprises 88.7% silver particles and 11.3% polymer solids.

The Young's modulus and hardness of the annealed silver wire at 300°C were tested using a nanoindentation tester, as shown in **Fig. S12**. The silver wire is inlaid into the resin and polished. The Young's modulus is 45.72 GPa, 0.55 times that of the silver block. This is due to polymers in the silver material, resulting in a lower Young's modulus. The measured hardness obtained is 1.67 GPa. This hardness is 0.67 times higher than that of the silver block.

The adhesion test is carried out in accordance with the standard issued by the International Organization for Standardization (ISO 2409, paints and varnishes - cross-cut test). In this experiment, parallel silver wires were printed on the PI film with a width of 650 μm and a pitch of 350 μm. A 20 mm wide 3M tape is applied to the layer, acting on the 20 silver wires. Uniformly peel off the 3M adhesive tape using an electronic universal testing machine (TS E45.105) and record the adhesive force as shown in **Fig. S13**a. The adhesion at the initial point of tape separation was 3.42 N. The adhesive strength of the silver wire is greater than 0.17 N/mm. The printed silver wire is intact and not flaking, as shown in **Fig. S13**b and c. The adhesion of the silver wire to the substrate meets Class 0 standards.

A breakdown experiment has been performed to evaluate the stability of the printed spatial silver wire. **Fig. S15**a illustrates a printed silver wire with a linewidth of 20 μm and a length of 2 mm. The current density was measured by placing the silver wire in a direct current (DC) field.^1^ **Fig. S14** demonstrates that the current density increases as the applied electric field increases. The current density of the silver wire reaches 4×10^8^ A/m^2^ before breakdown when subjected to a thermal treatment temperature of 500 °C. The saturation current density of the silver wire decreases as the annealing temperature decreases. **Fig. S15**b illustrates that the silver wire experiences failure at its midpoint when subjected to high currents. The silver wire at the failure site was melted and transformed into multiple spherical structures at elevated temperatures.

**Fig. S16**a illustrates a schematic of a 5×5 array of transversal and longitudinal conductive interconnects printed on a polyimide (PI) film. The printing process utilized a thermal plate temperature of 100 °C and a nozzle with an inner diameter of 50 μm. The printing speed of the planar wire was set at 0.2 mm/s with an air pressure of 4 kPa. The printing speed of spatial wire was set at 40 μm/s with an air pressure of 2.5 kPa. The circuit was thermally treated at 150 °C for 30 minutes. The light-emitting diodes (LEDs) were fixed to the circuit (**Fig. S16**b).

Bending tests are performed in accordance with the standards developed by the International Electronics Manufacturing Initiative (IPC-6013, Flexible Printed Circuits Qualification and Performance Specification). Test parameters are 200 bending cycles, a 6 mm bending radius, and a 5 mm/s bending rate, *i.e.*, the test fixture moves speed, as shown in **Fig. S17**. The bending point is guaranteed to be in the middle of the circuit. The slewing stroke is ±45° respectively. The continuity of the circuit can still be maintained after continuous testing.

As illustrated in **Fig. S18**a, an infrared camera captured the heating of the silver wire at different voltages. The temperature of the silver wire gradually increases as the voltage increases. This is because the output power of the silver wire increases with increasing current. As shown in **Fig. S18**b, the resistance of the silver wire increases with increasing temperature. Therefore, the heating power of the silver wire does not increase exponentially with increasing voltage.

The process for circuit preparation is depicted in **Fig. S19**. The PI flexible substrate was prepared to match the size of the circuit. The printing speed for planar surfaces was set at 0.3 mm/s with an air pressure of 5 kPa. The 3D architectural printing speed was 40 μm/s with an air pressure of 2.5 kPa. **Fig. S19**b depicts a structural diagram of the printed circuit. The printed circuit was thermally treated at 150 °C. Electronic components required for printed circuits were pasted onto the circuit with silver paste, as depicted in **Fig. S19**c.

3D Interconnect is an advanced electronics packaging and manufacturing technology that vertically stacks multiple semiconductor chips or electronic devices and forms a compact 3D structure through exemplary electrical connections, mainly including technologies such as wire bonding^2,3^ and through-silicon via (TSV)^4-6^. Wire bonding uses a capillary or wedge tool to connect wires to the pads of the chip. It is currently the most widely used packaging technology due to the advantages of a simple process, low cost, and easy maintenance^7,8^. Wire bonding, however, on flexible substrates requires special pad materials and higher temperatures to achieve good adhesion. Above all, the pressure of a capillary or wedge tool deforms the flexible substrate, resulting in a reduced interfacial force between the bonding wire and the bonding pad^9,10^. TSV uses etching to achieve vertical electrical interconnections from wafer to wafer. Its benefits include shorter interconnect lengths, lower latency, and higher energy efficiency^11^. The mismatch between the expansion coefficients of the filler materials copper and silicon can lead to warpage of the wafers and heat dissipation issues, which are also significant challenges^12,13^. The TSV is difficult to use due to the poor thermal stability and conductivity of flexible substrates, and it is currently used only on rigid substrates^14^.

We compare the technical differences between printing photosensitive resins, ceramics, and metals. 3D printing photosensitive resin is a vat photopolymerization (VP) technology. Photoinitiators react in the resin when exposed to UV light and catalyze the binding of oligomers and monomers to form polymer chains, which cross-link with other polymer chains, leading to curing^15,16^. VP technology has been widely studied for its fast-forming speed and high printing accuracy. 3D printing photosensitive resin is challenged by insufficient mechanical properties and poor material heat resistance due to the inhomogeneous crosslinked network structure formed by the rapid reaction process and the limited degree of curing^17-20^.

3D-printed metals are similar to ceramics in that they are primarily a build-up of powder particles. Selective laser sintering (SLS) directly uses a laser to selectively sinter powders to form 3D building blocks but suffers from high porosity and poor mechanical properties^21,22^. Binder jetting (BJ) is the selective deposition of liquid binder droplets into a powder bed. The powder fill density, however, is low, and solid parts are produced with relatively poor geometrical accuracy^21-23^. Slurries with photosensitive or heat-curing materials have been developed to ensure the molding of the material. Slurries with photosensitive or heat-curing materials to ensure material shaping have been developed for a wide range of applications^24^. As with VP technology, slurries with added light-curing materials can prepare 3D components layer by layer using stereolithography (SLA)^25,26^. Removing organic matter after annealing results in significant volume shrinkage, which tends to cause structural deformation or cracking. Direct writing (DW) would be cheaper, but the quality and resolution of the structure would be lower^27-29^. The omnidirectional and unsupported printing properties of DW are advantageous in fabricating linear structures^30-32^. DW on metallic materials has shown promising applications for interconnecting flexible circuits. Since DW is mainly extruded by the needle, the printing accuracy is affected by the inner diameter of the needle.


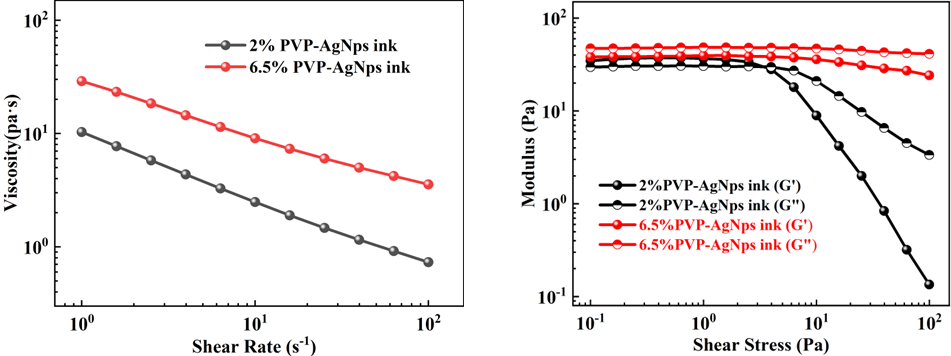


**Fig. S1.** The rheological properties of silver ink were evaluated. (a) Viscosity plotted against shear rate for the PVP-AgNPs ink. (b) Storage and loss modulus presented as a function of shear stress for the PVP-AgNPs ink.


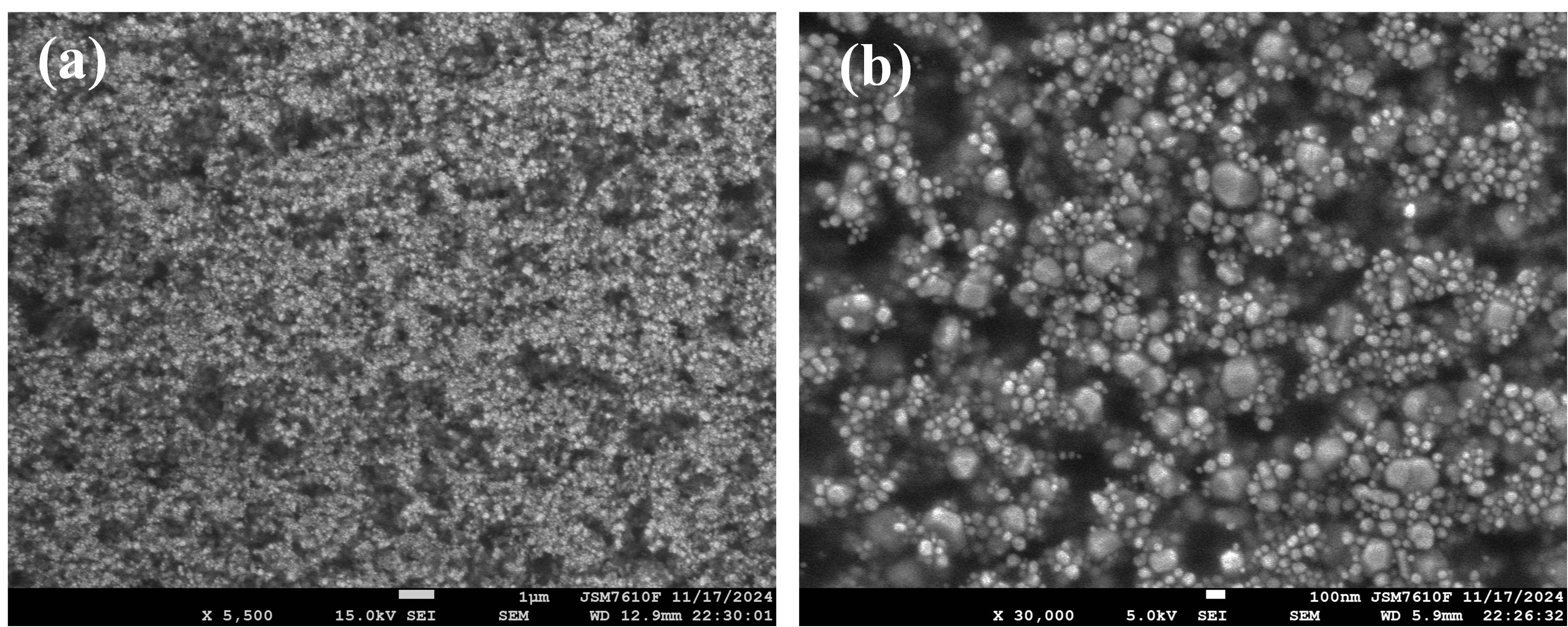


**Fig. S2**. SEM images of silver ink before thermal treatment.


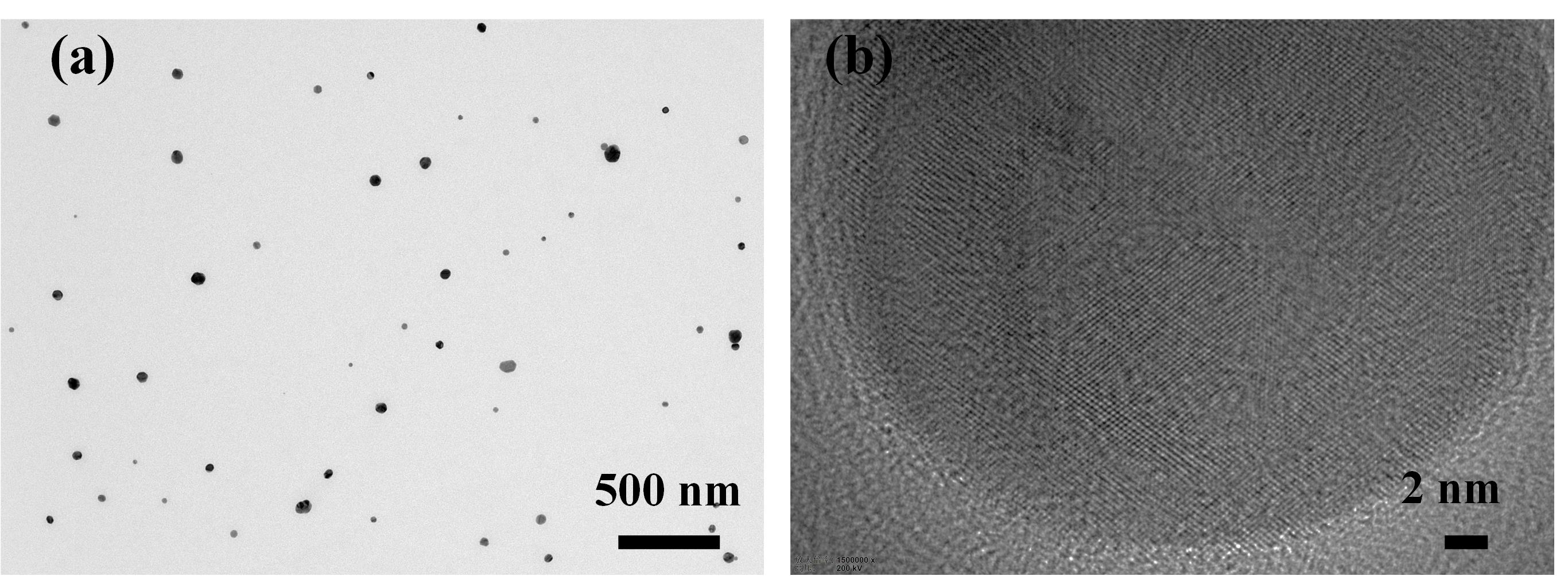


**Fig. S3**. TEM images of AgNPs.


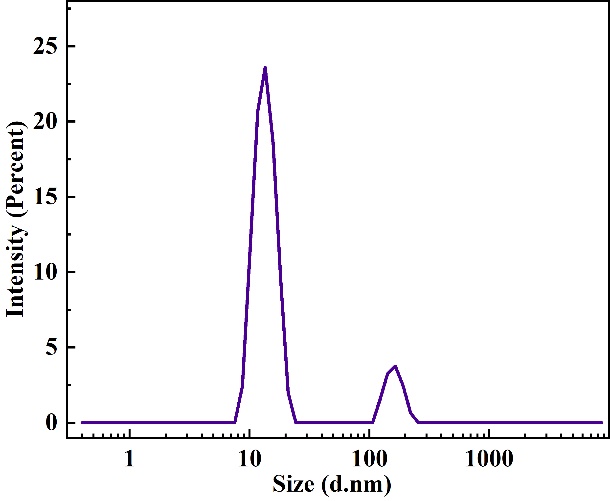


**Fig. S4**. The particle size ratio of AgNPs.


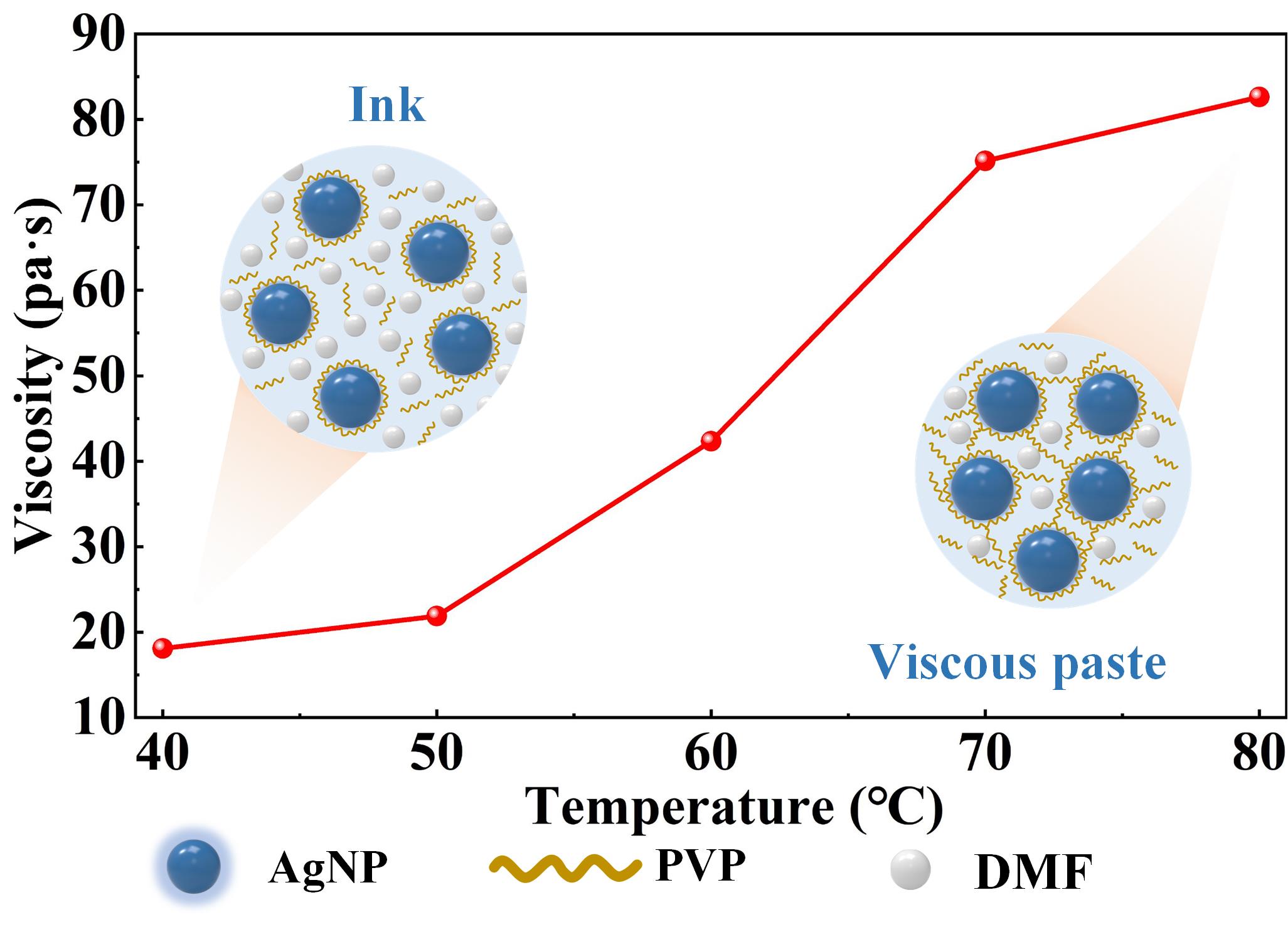


**Fig. S5.** Viscosity changes of ink at different temperatures.


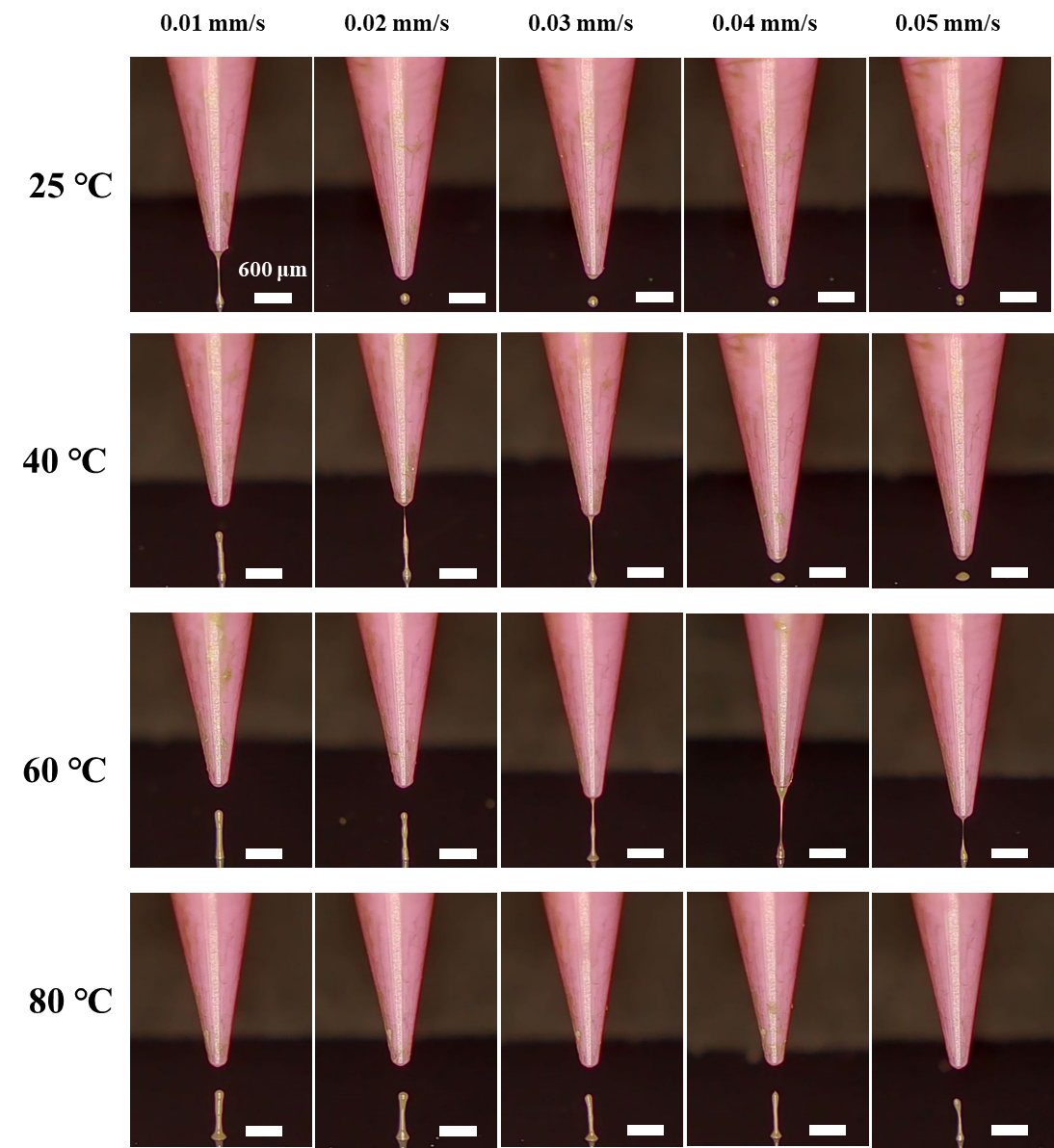


**Fig. S6.** Pictures of printing results with different temperatures and different needle lift speeds.


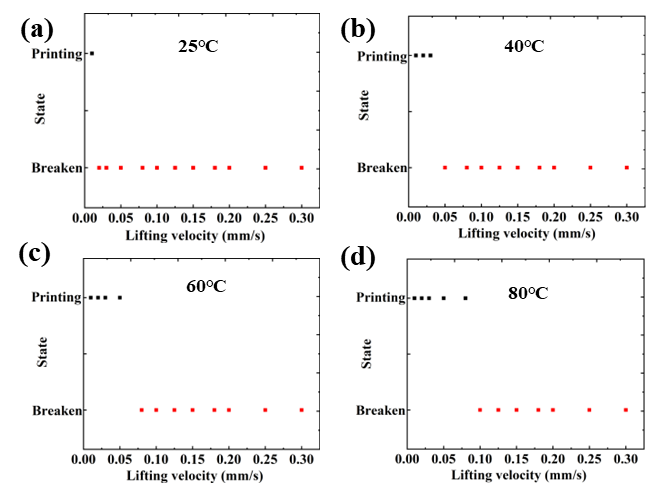


**Fig. S7.** The impact of lifting velocity on the break of silver wire at various temperatures is examined. The state of silver filaments at different speeds at 25 °C (a), 40 °C (b), 60 °C (c) and 80 °C (d).


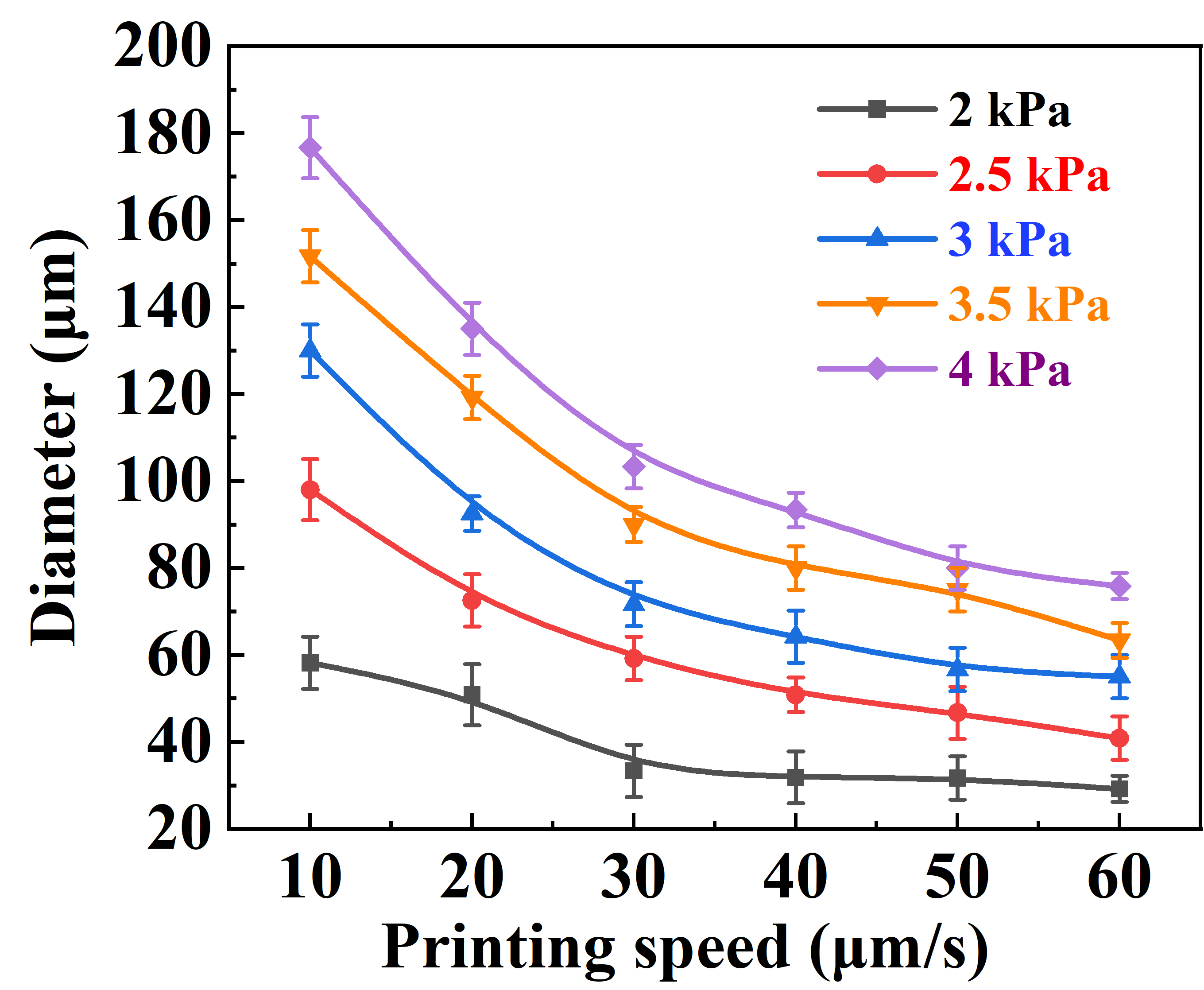


**Fig. S8.** Variation in wire width of silver columns printed at different air pressures and speeds.


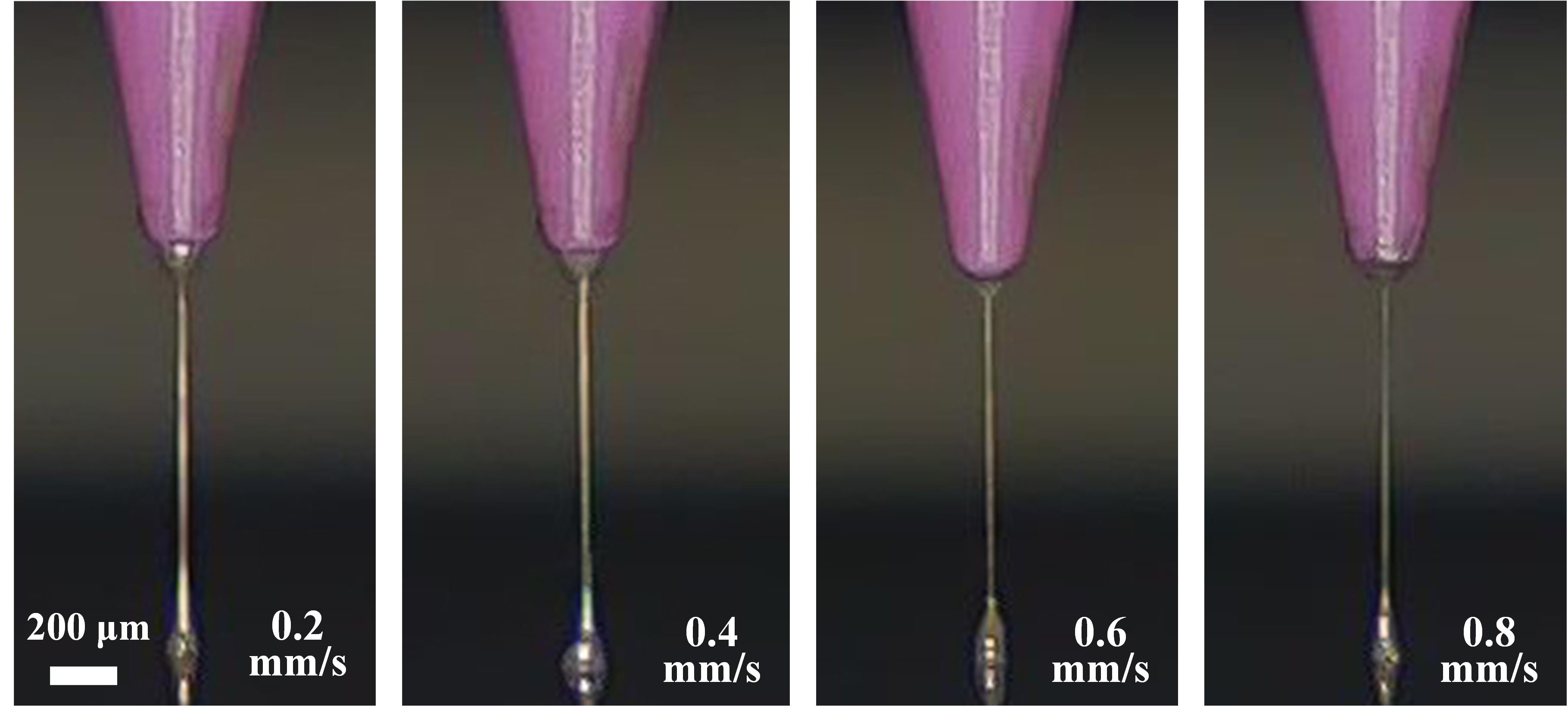


**Fig. S9.** Variable speed printing silver wire construction.


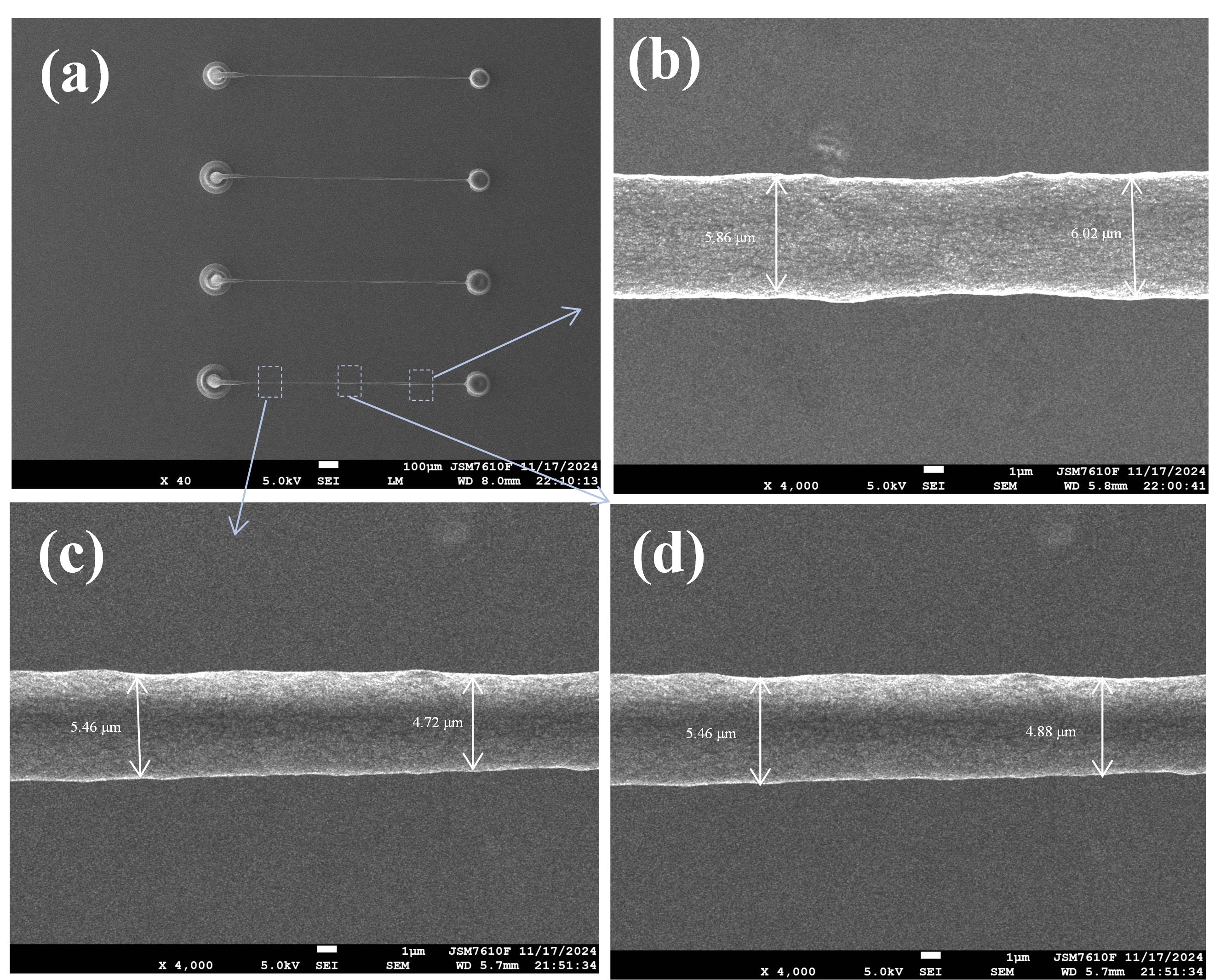


**Fig. S10.** Printed silver wires and line widths in different positions.

**Table S1** Line width at different positions with silver wire tolerances (μm)

| Wires | 1 | 2 | 3 | 4 | 5 | 6 | Average | Tolerance |
| --- | --- | --- | --- | --- | --- | --- | --- | --- |
| W-1 | 5.86 | 6.02 | 5.46 | 4.72 | 5.46 | 4.88 | 5.40 | 1.30 |
| W-2 | 6.13 | 6.07 | 5.19 | 4.97 | 4.73 | 5.34 | 5.41 | 1.40 |
| W-3 | 5.73 | 6.22 | 4.93 | 5.91 | 4.81 | 5.56 | 5.52 | 1.41 |
| W-4 | 5.86 | 5.91 | 5.62 | 4.67 | 4.52 | 5.02 | 5.27 | 1.24 |


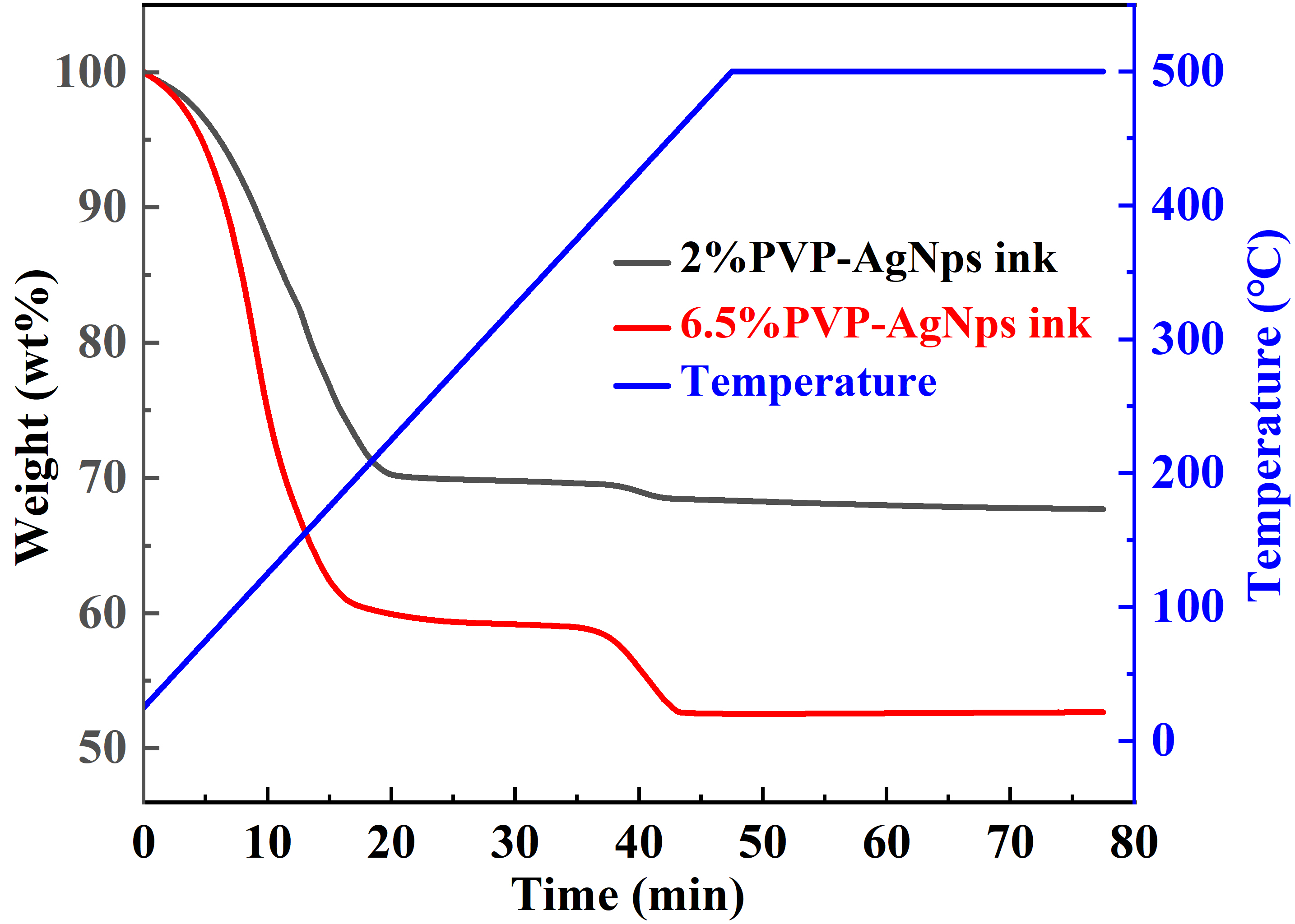


**Fig. S11.** Thermal treatment curves of silver inks with different PVP contents.


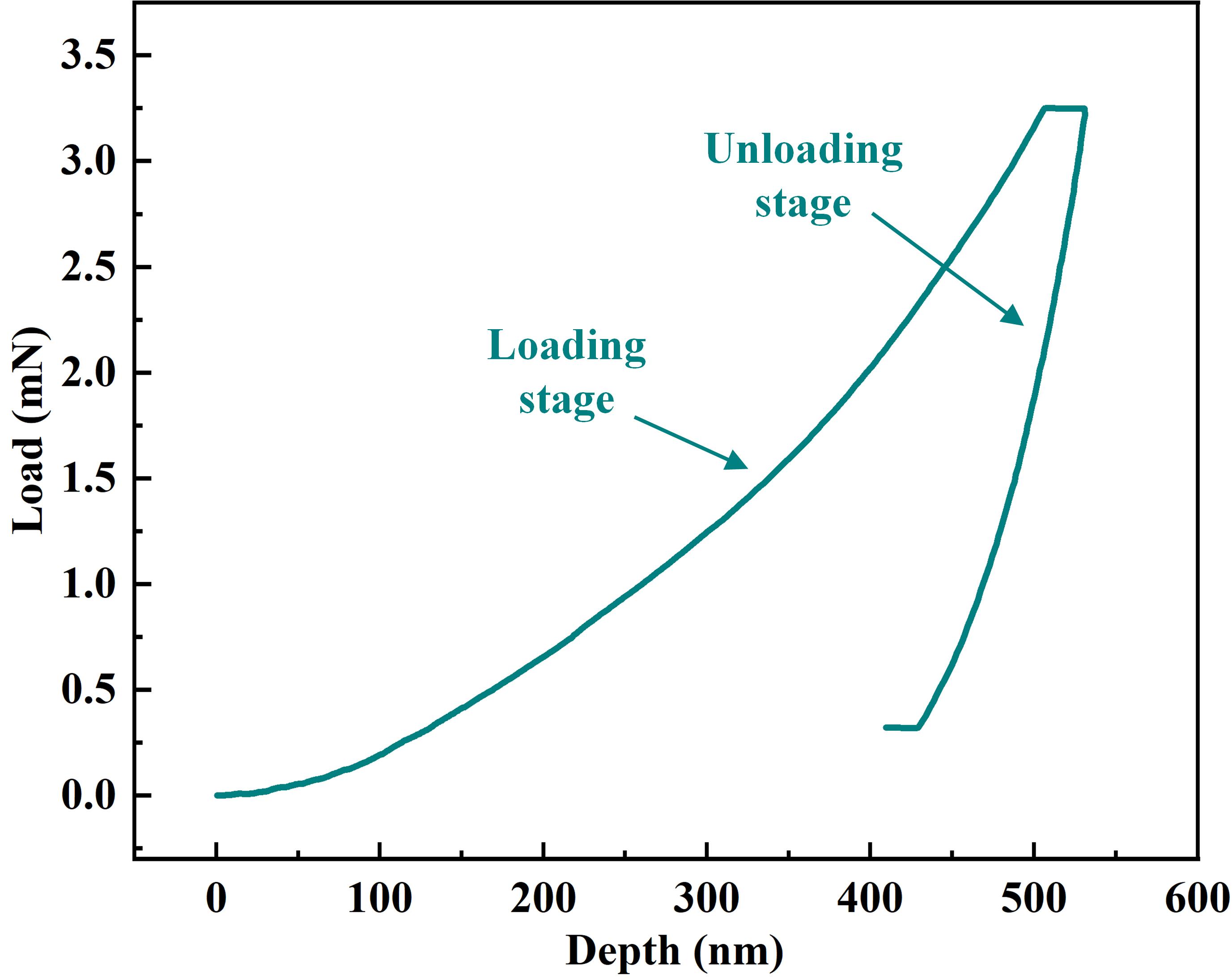


**Fig. S12.** Relationship between force load and indentation depth.

**Table S2** Young's modulus and hardness of silver ink after annealing

| Points | Depths  (nm) | Maximum load  (mN) | Young's modulus  (GPa) | Hardness  (GPa) |
| --- | --- | --- | --- | --- |
| 1 | 517.81 | 3.31 | 50.73 | 1.89 |
| 2 | 506.31 | 3.25 | 49.95 | 2.18 |
| 3 | 515.91 | 4.07 | 41.22 | 1.36 |
| 4 | 507.68 | 4.52 | 39.51 | 1.61 |
| 5 | 535.18 | 4.84 | 47.20 | 1.32 |
| Average | 516.58 | 4.00 | 45.72 | 1.67 |


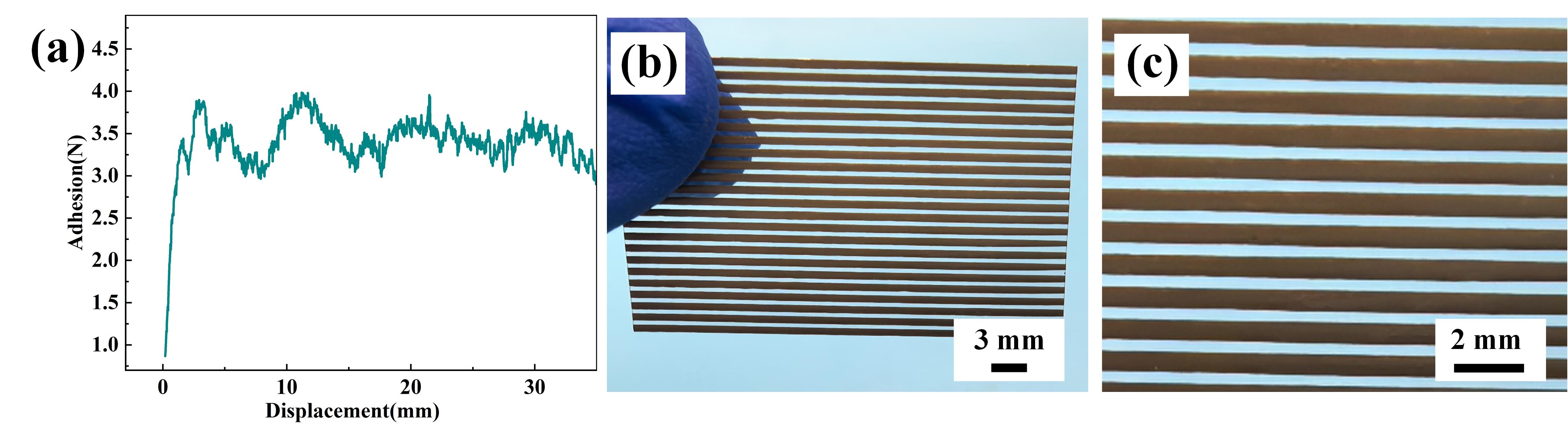


**Fig. S13.** Bonding test of silver wires. (a) Relationship between adhesion and displacement. (b) Silver wires were printed on the PI. (c) Localized enlargement of the silver wire.


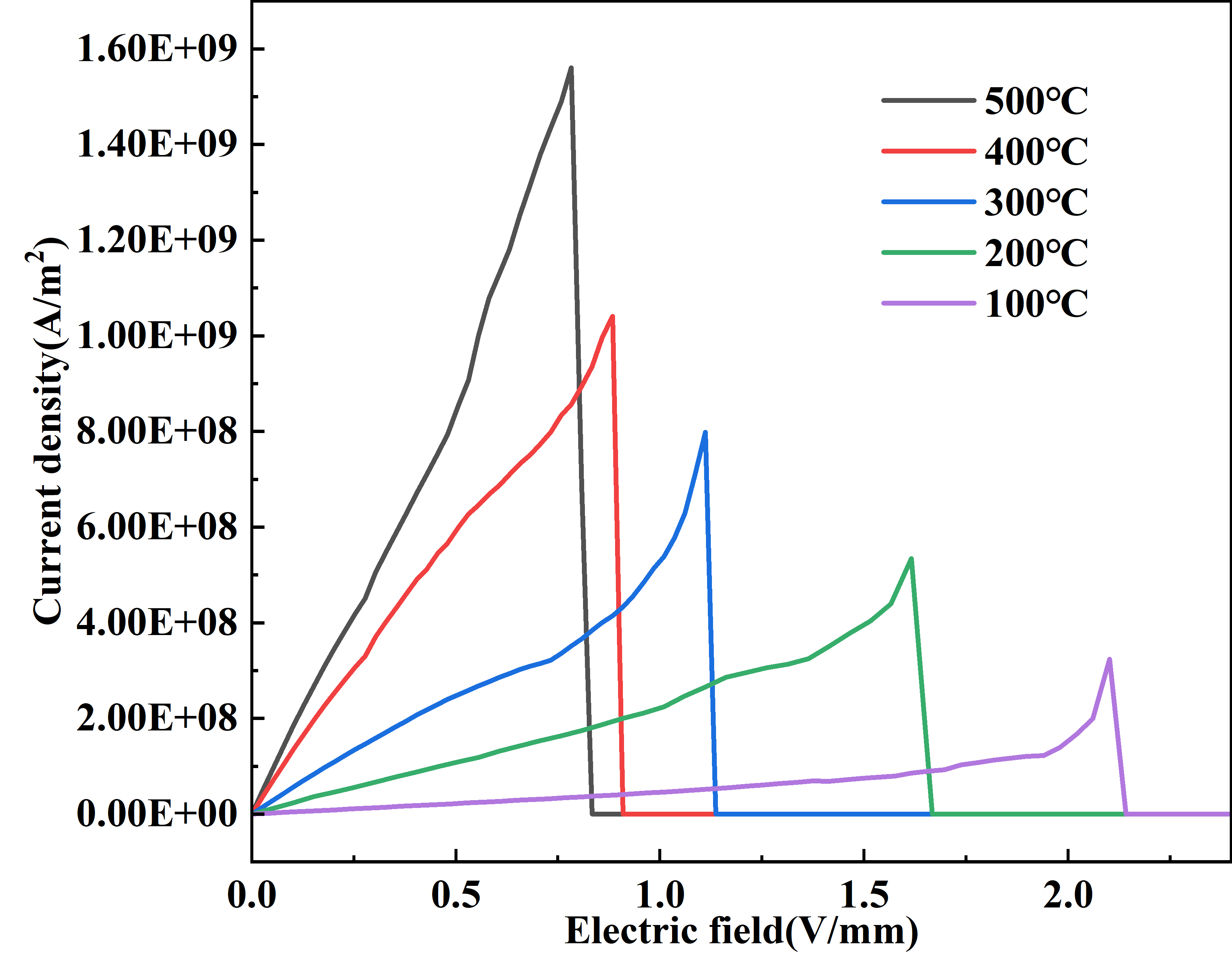


**Fig. S14.** Bias current density and response of silver wire at different thermal treatment temperatures.


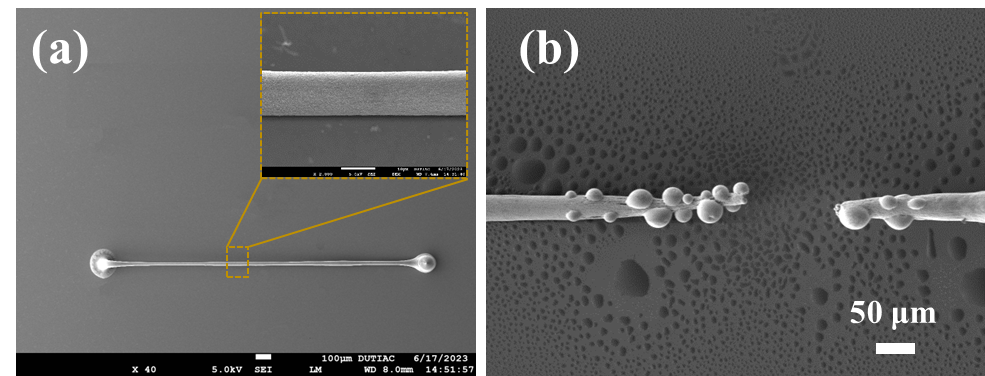


**Fig. S15.** Scanning electron microscopy (SEM) image of the printed silver wire. (a) The printed 3D silver wire. The inset is a localized SEM characterization of a silver filament. (b) Silver conductors that fuse under high currents.


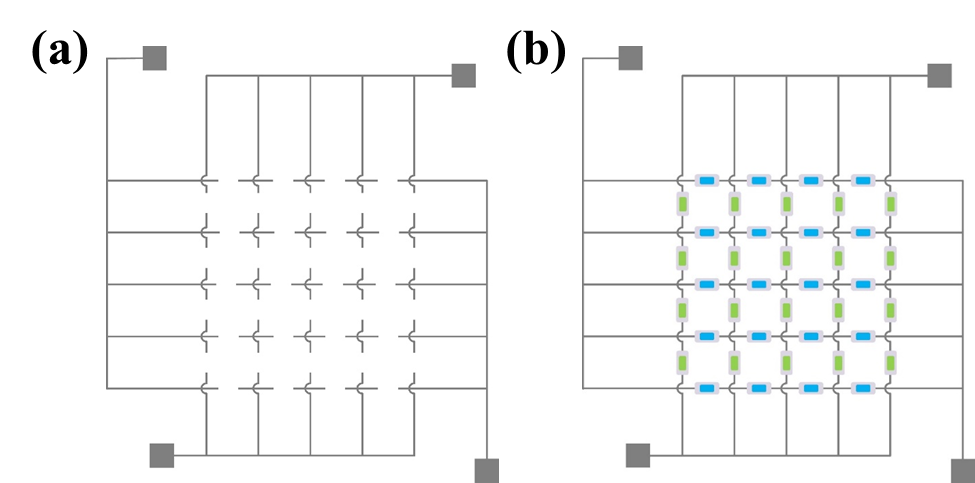


**Fig. S16.** The schematic of the LED array is shown. (a) Array circuits printed on PI film. (b) Fixing the LED to the printed circuit.


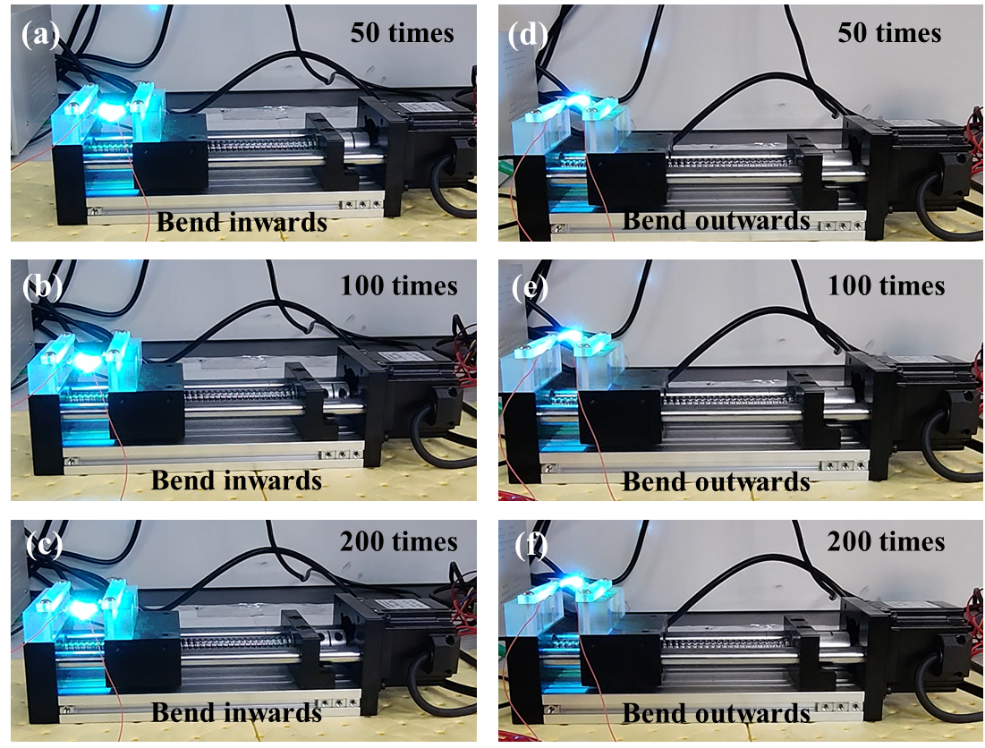


**Fig. S17.** Flexible LED array circuit bending test.


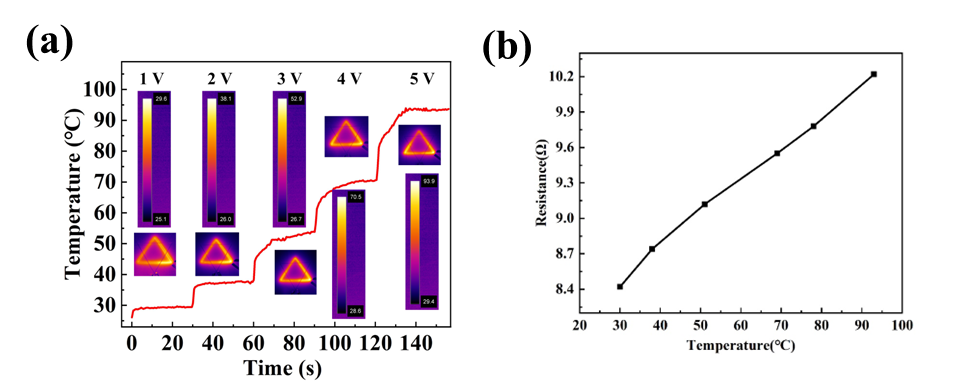


**Fig. S18.** (a) The relationship between temperature versus applied voltage curve for silver wire. (b) The relationship between the resistance value of the silver wire and the temperature change.


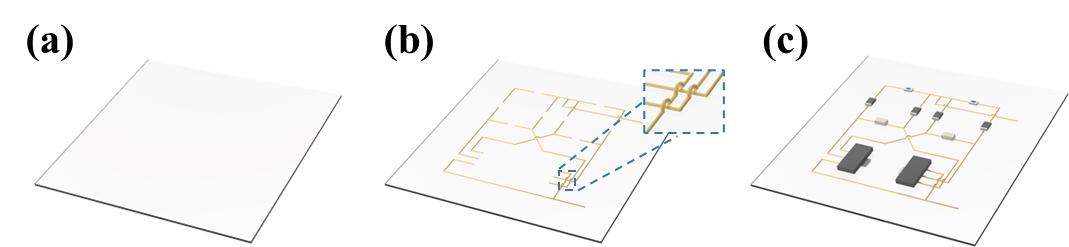


**Fig. S19.** The manufacturing process for multivibrator circuit.

**Table S3.** Different methods of printing 3D conductive structure

| **Reference** | **Materials** | **Viscosity** | **Needle diameter** | **printing speed** | **linewidth** | **Conductivity** |  |  |  |
| --- | --- | --- | --- | --- | --- | --- | --- | --- | --- |
| Seol *et al.*^33^ | AgNps/PAA | 7 mPa·s | 5-15 μm | 0.01 mm/s | 5-10 μm | 1×10^-4^ Ω·cm |  |  |  |
| Seol *et al.*^34^ | | | MWNT/PVP | 12.64 mPa·s | 12 μm | 0.075 mm/s | 9 μm | 4×10^-2^ Ω·cm |  |
| **This work** | | | | AgNps/PVP | 29 Pa·s | 50 μm | 0.01-1 mm/s | 4-180 μm | 2.5×10^-5^ Ω·cm |
| Lewis *et al.*^30^ | | AgNps/PAA | 100 Pa·s | 1-100 μm | 0.02-0.5 mm/s | 1-100 μm | 5.2×10^-5^ Ω·cm |  |  |
| Lan *et al.*^35^ | AgNps (ST201S1G01A) | 1000 Pa·s | 500 μm | 0.7 mm/s | 80-560 μm | N/A |  |  |  |
| Lee *et al*.^36^ | AgNps/MWNT | 2.05×10^5^ Pa·s | 108-210 μm | 2-50 mm/s | 100 μm- mm | 1.5×10^-4^ Ω·cm |  |  |  |

1 Zhao, J. *et al.* Electrical breakdown of nanowires. *Nano Lett* **11**, 4647-4651, (2011).

2 Xie, L. *et al.* State-of-the-art of the bond wire failure mechanism and power cycling lifetime in power electronics. *Microelectronics Reliability* **147**, (2023).

3 Zhang, Y. *et al.* Research Progress of Palladium-Plated Copper Bonding Wire in Microelectronic Packaging. *Micromachines* **14**, (2023).

4 Wang, J. *et al.* A Short Review of Through-Silicon via (TSV) Interconnects: Metrology and Analysis. *Applied Sciences* **13**, (2023).

5 Vethamuthu Edward Alaises, R. S. *et al.* Novel BIST Solution to Test the TSV Interconnects in 3D Stacked IC’s. *Electronics* **12**, (2023).

6 Wang, Z. Microsystems using three-dimensional integration and TSV technologies: Fundamentals and applications. *Microelectronic Engineering* **210**, 35-64, (2019).

7 An, B. *et al.* A Review of Silver Wire Bonding Techniques. *Micromachines* **14**, (2023).

8 Chauhan, P. *et al.* Copper Wire Bonding Concerns and Best Practices. *Journal of Electronic Materials* **42**, 2415-2434, (2013).

9 Ji, B. *et al.* Flexible Optoelectric Neural Interface Integrated Wire-Bonding $\mu$ LEDs and Microelectrocorticography for Optogenetics. *IEEE Transactions on Electron Devices* **64**, 2008-2015, (2017).

10 Li, Q. *et al.* Review of the Failure Mechanism and Methodologies of IGBT Bonding Wire. *IEEE Transactions on Components, Packaging and Manufacturing Technology* **13**, 1045-1057, (2023).

11 Lau, J. H. Recent Advances and Trends in Advanced Packaging. *IEEE Transactions on Components, Packaging and Manufacturing Technology* **12**, 228-252, (2022).

12 Dahiya, R. S. *et al.* Bendable Ultra-Thin Chips on Flexible Foils. *IEEE Sensors Journal* **13**, 4030-4037, (2013).

13 Gambino, J. P. *et al.* An overview of through-silicon-via technology and manufacturing challenges. *Microelectronic Engineering* **135**, 73-106, (2015).

14 Dahiya, A. S. *et al.* Printed Interconnects for Heterogeneous Systems Integration on Flexible Substrates. *Advanced Materials Technologies*, (2024).

15 Gao, W. *et al.* Dual-curing polymer systems for photo-curing 3D printing. *Additive Manufacturing* **85**, (2024).

16 Liu, W. *et al.* Research Progress of Self-Healing Polymer for Ultraviolet-Curing Three-Dimensional Printing. *Polymers* **15**, (2023).

17 Fei, J. *et al.* Progress in Photocurable 3D Printing of Photosensitive Polyurethane: A Review. *Macromolecular Rapid Communications* **44**, (2023).

18 Chekkaramkodi, D. *et al.* Review of vat photopolymerization 3D printing of photonic devices. *Additive Manufacturing* **86**, (2024).

19 Moore, L. M. J. *et al.* Polybutadiene Click Chemistry: A Rapid and Direct Method for Vat Photopolymerization. *ACS Applied Polymer Materials* **5**, 9138-9146, (2023).

20 Tessanan, W. *et al.* Development of Photosensitive Natural Rubber as a Mechanical Modifier for Ultraviolet-Curable Resin Applied in Digital Light Processing-Based Three-Dimensional Printing Technology. *ACS Omega* **6**, 14838-14847, (2021).

21 Zhao, D. *et al.* A critical review of direct laser additive manufacturing ceramics. *International Journal of Minerals, Metallurgy and Materials* **31**, 2607-2626, (2024).

22 Papazoglou, E. L. *et al.* On the Modeling and Simulation of SLM and SLS for Metal and Polymer Powders: A Review. *Archives of Computational Methods in Engineering* **29**, 941-973, (2021).

23 Sing, S. L. *et al.* Direct selective laser sintering and melting of ceramics: a review. *Rapid Prototyping Journal* **23**, 611-623, (2017).

24 Sun, J. *et al.* A review on additive manufacturing of ceramic matrix composites. *Journal of Materials Science & Technology* **138**, 1-16, (2023).

25 Ferraro, V. *et al.* Recent Advances of Transition Metal Complexes for Photopolymerization and 3D Printing under Visible Light. *Advanced Functional Materials* **34**, (2023).

26 Xiaobo *et al.* 3D Printing of Complex‐type SiOC Ceramics Derived From Liquid Photosensitive Resin. **4**, 6862-6869, (2019).

27 Hou, Z. *et al.* Direct Ink Writing of Materials for Electronics-Related Applications: A Mini Review. *Frontiers in Materials* **8**, (2021).

28 del-Mazo-Barbara, L. *et al.* Rheological characterisation of ceramic inks for 3D direct ink writing: A review. *Journal of the European Ceramic Society* **41**, 18-33, (2021).

29 Feng, J. *et al.* Direct-ink-writing 3D Printing of Ceramic-based Porous Structures: a Review. *Journal of Inorganic Materials* **38**, (2023).

30 Ahn, B. Y. *et al.* Omnidirectional printing of flexible, stretchable, and spanning silver microelectrodes. *Science* **323**, 1590-1593, (2009).

31 Skylar-Scott, M. A. *et al.* Laser-assisted direct ink writing of planar and 3D metal architectures. *Proc Natl Acad Sci U S A* **113**, 6137-6142, (2016).

32 Zhao, Y. *et al.* 3D printing of unsupported multi-scale and large-span ceramic via near-infrared assisted direct ink writing. *Nature Communications* **14**, (2023).

33 Lee, S. *et al.* Three-dimensional Printing of Silver Microarchitectures Using Newtonian Nanoparticle Inks. *ACS Applied Materials & Interfaces* **9**, 18918-18924, (2017).

34 Kim, J. H. *et al.* Three-Dimensional Printing of Highly Conductive Carbon Nanotube Microarchitectures with Fluid Ink. *ACS Nano* **10**, 8879-8887, (2016).

35 Sun, P. *et al.* Directly Printed Interconnection Wires between Layers for 3D Integrated Stretchable Electronics. *Advanced Materials Technologies* **7**, (2022).

36 Lee, B. *et al.* Omnidirectional printing of elastic conductors for three-dimensional stretchable electronics. *Nature Electronics* **6**, 307-318, (2023).

**Supporting Movie S1:** Planar and 3D interconnects of different line widths.

**Supporting Movie S2:** Printing crossover and isolation circuits with different line widths.

**Supporting Movie S3:** Printing silver columns at varying air pressures.

**Supporting Movie S4:** Prints continuous 3D filament structures with different line widths.

**Supporting Movie S5:** Printing spanning 3D interconnects with different line widths.

**Supporting Movie S6:** Flexible crossover LED circuit experiment.

**Supporting Movie S7:** Flexible thermal imaging display experiment.

**Supporting Movie S8:** Flexible multivibrator circuit experiment.
